# The Effect of Macromonomer Surfactant Microstructure on Aqueous Polymer Dispersion and Derived Polymer Film Properties

**DOI:** 10.1021/acs.biomac.4c00292

**Published:** 2024-06-11

**Authors:** Ingeborg Schreur-Piet, Johan P.A. Heuts

**Affiliations:** Department of Chemical Engineering & Chemistry and Institute for Complex Molecular Systems, Eindhoven University of Technology, P.O. Box 513, 5600 MB Eindhoven, The Netherlands

## Abstract

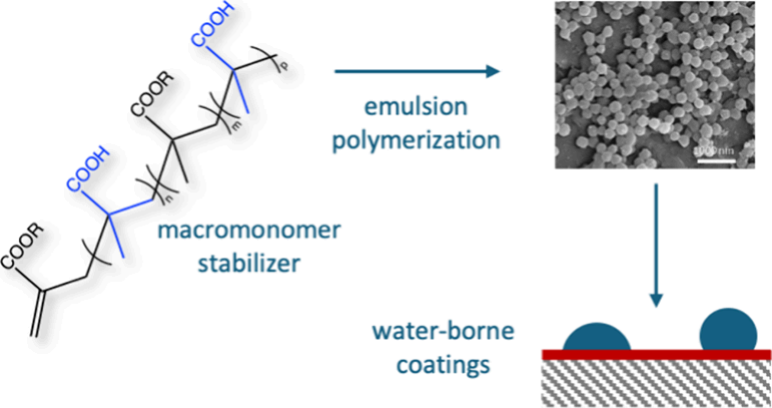

Water-borne coatings were prepared from poly(methyl methacrylate-*co*-butyl acrylate) latexes using different methacrylic acid containing macromonomers as stabilizers, and their physical properties were determined. The amphiphilic methacrylic acid macromonomers containing methyl, butyl, or lauryl methacrylate as hydrophobic comonomers were synthesized via catalytic chain transfer polymerization to give stabilizers with varying architecture, composition, and molar mass. A range of latexes of virtually the same composition was prepared by keeping the content of methacrylic acid groups during the emulsion polymerization constant and by only varying the microstructure of the macromonomers. These latexes displayed a range of rheological behaviors: from highly viscous and shear thinning to low viscous and Newtonian. The contact angles of the resulting coatings ranged from very hydrophilic (<10°) to almost hydrophobic (88°), and differences in hardness, roughness, and water vapor sorption and permeability were found.

## Introduction

**Scheme 1:**
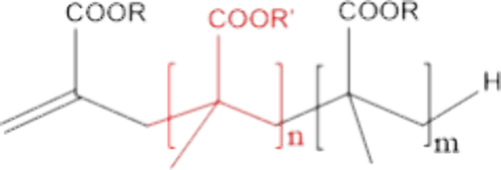
General Structure of a Macromonomer of a Methacrylate and MAA, R, and R′ Representing H or an Alkyl Group

Colloidal polymer particles have been studied over the past several decades, and their applications have received an increasing attention in various fields, ranging from various biomedical applications, such as medical imaging,^1^ drug delivery systems,^2−7^ biosensors,^8−10^ biofilms,^10−12^ and antibacterial coatings,^12−16^ and applications such as pressure sensitive adhesives^17−19^ and protective^20−23^ and antifouling coatings.^24,25^ These colloidal polymer particles are often synthesized via emulsion polymerization and can be functionalized using dedicated monomers and surfactants. The surfactants used in emulsion polymerization not only control the colloidal stability of the particles in the polymer dispersion (i.e., the latex) but their composition and microstructure (block copolymers, branched copolymers, polymer brushes) can also have a large influence on the physical properties of the latex and the final polymer film (or coating).^1,26−28^ In general, most used surfactants are not covalently bound to the colloidal particles and can diffuse from the particles into the environment or from the bulk to the film surface (and potentially desorb). This is especially undesirable in the case of biomedical applications, but also more generally, this may negatively affect the polymer film properties, such as adhesion, surface polarity, smoothness, and gloss.^29−31^ To prevent desorption of surfactants from the particle surfaces or migration from the bulk polymer film (and potential leaching into the environment), reactive surfactants, which are chemically bound to the polymer particles, can be used,^31−41^ and those containing propenyl end-groups are promising candidates.^42−44^ Methacrylic oligomers containing these end-groups (see Scheme 1) are readily prepared via catalytic chain transfer polymerization (CCTP),^45−52^ and their application in emulsion polymerization has been described previously.^35,42,53−56^ Amphiphilic block macromonomers can be obtained by chain extension via sulfur-free reversible addition–fragmentation chain transfer^44,57−60^ or used directly in an emulsion polymerization to form in situ amphiphilic copolymers, in a mechanism similar to what is commonly known as polymerization-induced self-assembly.^1,61−65^ In an earlier work, we synthesized macromonomers via CCTP, which were successfully used as reactive surfactants in an emulsion polymerization.^66−68^ These macromonomers were composed of methacrylic acid (MAA) and methyl methacrylate (MMA), butyl methacrylate (BMA), lauryl methacrylate (LMA), or butyl acrylate (BA) and used in the emulsion polymerization of methyl methacrylate and/or butyl acrylate. By varying the composition, chain length, and concentration of the macromonomers, we were able to tune the particle size, molar mass, and rheological behavior of the latexes. We found that pMMA latexes were best stabilized by statistical MAA macromonomers containing BMA and LMA as comonomers, and these latexes showed a small yield stress and shear thinning behavior, properties which are useful for binders in, e.g., waterborne paints.^69^

In this work, we selected earlier synthesized macromonomers with different architectures and compositions to prepare latexes and coatings to investigate the influence of the used macromonomer structure on the latex and film properties while keeping the amount of MAA groups (0.025 mol) of the macromonomer and the overall latex composition constant. Although the systems we investigate are not directly aimed at biomedical applications, we expect the results to be of general relevance to any emulsion polymerization using reactive polymeric surfactants.

## Experimental Section

### Materials

MMA and BA were obtained from Sigma-Aldrich (99%) and passed over a column of inhibitor remover (Sigma-Aldrich). Potassium persulfate (KPS, p.a.) and sodium carbonate (dehydrated, p.a.) were purchased from Merck and used as received. Macromonomers were synthesized and characterized as part of previously published work,^66−68^ and their main characteristics are summarized in Table 1.

**Table 1:** Characteristics of Macromonomeric Surfactants^66−68^

macromonomer^a^	type	*DP*_n_^c^	*F*_M_^d^	*T*_g_^e^ (°C)
*s*-L_204–18_	p(MAA-*stat-*LMA)	222	0.08	131
*s*-L_5–1_^b^		5	0.10	1
*s*-B_297–34_	p(MAA-*stat*-BMA)	331	0.10	161
*s*-B_3–1_		4	0.25	1
*s*-M_90–9_	p(MAA-*stat-*MMA)	99	0.09	172
*s*-M_30–10_		40	0.25	163
*b*-M_12–15_	p(MAA-*b-*MMA)	27	0.56	65
*h*-MAA_350_	pMAA	350	0	182
*h*-MAA_8_		8	0	42

a Notation: “L_*n*–*m*_” = MAA_*n*_-*co-*LMA_*m*_ “B_*n*–*m*_” = MAA_*n*_-*co-*BMA_m_, or “M_*n*–*m*_” = MAA_*n*_-*co-*MMA_*m*_; subscript *n*–*m* indicates the number of monomer units of both monomers; “*s*”, “*b*” or “*h*” = statistical, block, or homopolymer, respectively.

b *s*-L_5–1_ was used for clarity reasons; *s*-L_4.5–0.5_ would be a better reflection of the actual composition.

c Number-average degree of polymerization estimated from ^1^H NMR.

d *F*_M_ = mole fraction of hydrophobic comonomer in macromonomer estimated from ^1^H NMR, standard error ca. 5%.

e Glass transition temperature as measured using DSC.

### Preparation of Latexes

Emulsion polymerizations were carried out under an argon atmosphere in a standard 250 mL baffled jacketed thermostated glass reactor, equipped with a mechanical four-bladed turbine stirrer. For semibatch emulsion polymerizations, the reactor was charged with 75 g of deionized water, buffer, macromonomer, and 5 g of monomers (i.e., 9 wt % of the overall monomer content), stirred at 360 rpm, purged with argon for 30 min, and subsequently heated to 60 °C (see Table 2 for the standard recipe). Five minutes after reaching a constant temperature, a 4.1 × 10^–3^ M aqueous KPS solution (10 mL of water containing 0.1 g of KPS) was injected to start the polymerization.

**Table 2:** Standard Recipe of a Semi-batch Emulsion Polymerization of MMA and BA Using Different MAA (*co*)-Macromonomers as Surfactants^b^

ingredient	amount
water	85 g
Na_2_CO_3,_ buffer	1.4 g (1.3 × 10^–1^ M)
macromonomer	Variable; constant MAA content of 0.025 mol
MMA/BA (1:1 weight ratio)	55 g^a^; final solids content is ca. 40 wt %
K_2_S_2_O_8_ (KPS), initiator	0.1 g (4.1 × 10^–3^ M)

a 9 wt % added initially and polymerized for 1 h at *T* = 60 °C, remaining 91 wt % added after seed time with a feed rate of 5 mL h^–1^ at *T* = 70 °C.

b Stirring rate at 360 rpm, start *pH*_0_ = 7.5.

Starting 1 h after initiation, the temperature was raised to 70 °C to increase the polymerization rate, and the remaining monomer (50 g) was added at a constant feeding rate of 4–5 mL h^–1^, resulting in starved-feed conditions. The mixture was left to react overnight (24 h) to maximize the final conversion; conversions of the monomers were determined gravimetrically. The resulting polymers were characterized using size exclusion chromatography (SEC), differential scanning calorimetry (DSC), and dynamic vapor sorption (DVS). The latexes were characterized using dynamic light scattering (DLS) and scanning and transmission electron microscopy (SEM, TEM) for particle sizes and distributions, electrophoresis for zeta potentials, and rheometry for viscosity and dynamic moduli measurements. For details of these characterization techniques, see the Supporting Information (SI).

### Preparation of Coatings

Thin films were cast directly from the latex onto a glass substrate using a doctor blade applicator with an opening of 120 μm fixed in an application device, which moved at a constant speed of 10 mm/s. The films were dried overnight at either 20 or 40 °C. After drying, the films were annealed at 60 °C for 5 days. Thick films for DVS analyses were prepared by casting latex in an aluminum mold and drying in a vacuum oven at 60 °C for 5 days, and thick films for dynamic mechanical analysis (DMA) were prepared by casting latex in a Teflon mold and drying at room temperature. The surfaces of all films were examined visually and investigated by atomic force microscopy (AFM) and SEM. Physical properties of the thin films, such as contact angle and interfacial tension, were measured using an OCA15Pro goniometer. Additionally, the König hardness, cross hatch adhesion, and gloss of these coatings were measured. For details of these characterization techniques, see the Supporting Information (SI).

## Results and Discussion

We will first discuss the emulsion polymerization, followed by a discussion of the characterization of the latexes, emulsion polymers, and polymer films.

### Synthesis and Analysis of Latexes and Emulsion Polymers

**Figure 1:**
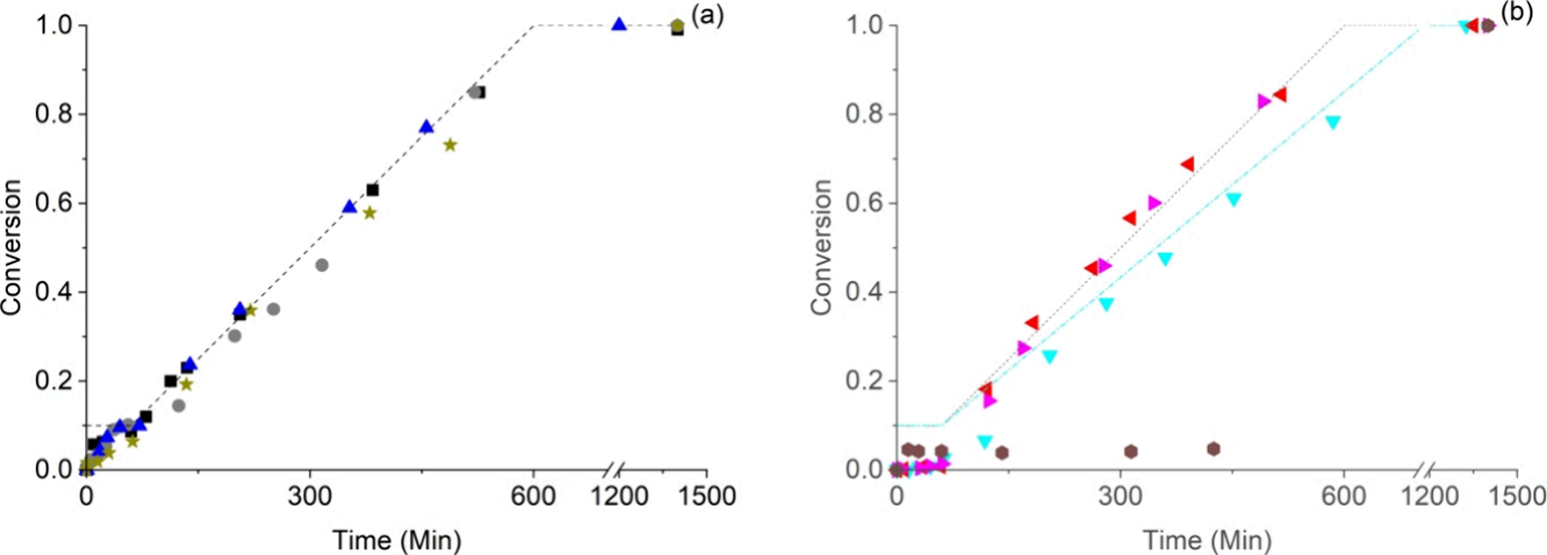
Overall monomer conversion versus time curves for the semibatch emulsion copolymerization of BA and MMA (1:1 w/w) with different macromonomers; (a) (box solid) *s-*L_204–18_; (circle solid) *s-*L_5–1_; (triangle up solid) *s-*B_297–34_; (diamond solid) *b-*M_12–15_ and (star solid) *h*-MAA_8_ with feed rate of 5 mL h^–1^; (b) (triangle left solid) *s-*M_90–9_; (triangle right solid) *s-*M_30–10_ and (hexagon solid) *h*-MAA_350_ with feed rate of 5 mL h^–1^ and (triangle down solid) *s-*B_3–1_ with a feed rate of 4 mL h^–1^. Initial charge of monomer 9 wt %, seed time 1 h; remaining 91 wt % fed (dashed line).

Latexes with an overall solids content of ∼40% were prepared using the different methacrylic acid macromonomers from Table 1 with a constant amount of MAA units in the reactor (0.025 mol). A typical graph of the overall monomer conversion and total amount of monomer fed for the starved-feed polymerizations is shown in Figure 1a. From this graph, it can be seen that during the seed stage (i.e., the first hour at 60 °C), the polymerization rate first increases rapidly, then levels off (see SI for more details), is constant during the following feed stage, and finally decreases due to a decreasing monomer concentration. The polymerization was started at 60 °C to have a comparable temperature during the seed stage as in our previous work;^66−68^ after 1 h, the reactor temperature was increased to 70 °C to obtain a faster polymerization rate to maintain starved-feed conditions during the entire polymerization. All polymerizations reached a final conversion of at least 99% overnight. The polymerizations using relatively hydrophilic surfactants show a low polymerization rate during the seed stage of the reaction; these curves are shown in Figure 1b. In these polymerizations, the used macromonomeric surfactants are initially not amphiphilic enough and need to react with more hydrophobic monomers to form in situ micelles and primary particles, as was also observed in our earlier work.^66−68^ However, in most cases, the usual polymerization rates were reached again after some induction time, except for the polymerization with the *h*-MAA_350_ macromonomer, which needed a seed time of more than 8 h. In all cases, no significant coagulation (~ irreversible aggregates) was observed after the polymerization, except for some minor coagulation around the stirrer and on the reactor wall (<3 wt %).

As in our previous work,^66−68^ we categorized the latexes according to their visual appearance into three types to facilitate the further discussion: type I is a colloidally stable liquid-like latex (low viscosity, containing no visible sediment/coagulum), type II is a (weakly) redispersible, flocculated latex, and type III is a highly viscous latex with a strong internal physical network structure and behaves like a solid in rest.

**Figure 2:**
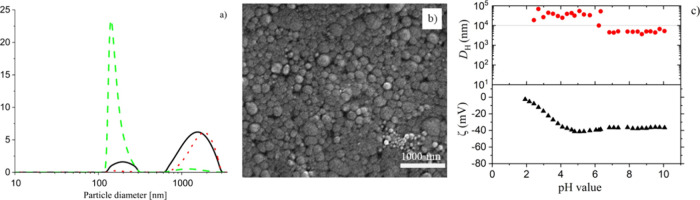
Overview of results from DLS and SEM of a typical type III latex: *s*L_l_; (a) PSD: intensity weighted (−); volume weighted (**..**) and number weighted (--) calculated using the CUMULANT model; (b) SEM image of diluted latex; (c) zeta potential (triangle up solid) and *D*_H_ (circle solid) as a function of the pH of diluted latex, which is adjusted to pH = 10 with 0.1 M NaOH solution and titrated with 0.01 M HCl solution; the horizontal blue line at *D*_H_ = 10^4^ nm (10 μm) indicates the upper instrument measuring threshold for particle sizes.

We determined the particle size distributions of the latexes using different techniques: DLS, SEM, and TEM. Furthermore, the zeta potential (ζ) of all latexes was measured at pH = 10 and for all latexes ζ and hydrodynamic diameter (*D*_H_) were measured as a function of pH. For the nomenclature of the latexes and their characteristics, see Table 3 (see the SI for PSD and the pH-dependence of *D*_H_ for each latex). Typically, for the type II and III latexes (*s*L_l_, *s*B_l_, *h*A_l_, and *h*A_s_), we observed broad particle size distributions (PSD) and high hydrodynamic diameters using DLS, (e.g., *D*_H_ for *s*L_l_ ≈ 2100 nm), with *D*_H_ being much larger than the number-averaged (*D*_n_), volume-averaged (*D*_v_), and intensity-averaged (*D*_I_) diameters. The results for type III latex (*s*L_l_) are shown in Figure 2.

**Table 3:** Characteristics of Synthesized Latexes Stabilized by MAA_*n*_-Based Macromonomers with Constant Added Amount of MAA in Reactor (0.025 mol)^a^

	macromonomer		particle size and zeta potential						rheology						
latex^b^	type^a^	wt %^c^	*D*_n,DLS_ (nm)^d^	*D*_H,DLS_ (nm)^e^	PDI_DLS_	*D*_n,SEM_ (nm)^d^	*D*_n,TEM_ (nm)^d^	ζ (mV)^f^	type^g^	η_min_^h^ (Pa s)^h^	η_max_ (Pa s)^h^	i	τ_min_^h^ (Pa)	*G*′ (Pa)^h^	*G*″ (Pa)^h^
*s*L_l_	*s-*L_204–18_	5.1	170	2079	0.33	173	-	–35	III	3.0 × 10^4^	3.7 × 10°	ST	2 × 10^1^	2 × 10^3^	1.1 × 10^3^
*s*L_s_	*s*-L_5–1_	5.7	74	144	0.22	66	69^f^	–44	I	3.5 × 10^–1^	1.0 × 10^–2^	ST	4 × 10^–4^	<1 × 10^–5^	9.8 × 10^–2^
*s*B_l_	*s*-B_297–34_	4.8	350	393	0.073	300	350	–43	II	2.0 × 10^2^	2.3 × 10^–1^	ST	3 × 10^–1^	9.9 × 10°	3.0 × 10^–1^
*s*B_s_	*s*-B_3–1_	7.1	299^$^	472^$^	0.19^$^	344	345	–66	I	1.4 × 10^–2*^	8.2 × 10^–3^	N	n.a	<1 × 10^–5^	8.2 × 10^–2^
*s*M_l_	*s*-M_90–9_	4.8	126	192	0.19	136	160	–33	I^#^	2.2 × 10^–2*^	2.1 × 10^–3^	N	n.a	<1 × 10^–5^	1.8 × 10^–1^
*s*M_s_	*s*-M_30–10_	6.0	184	325	0.22	152	155	–47	I^#^	2.0 × 10^–2*^	2.0 × 10^–3^	N	n.a	<1 × 10^–5^	1.8 × 10^–1^
*b*M	*b*-M_12–15_	9.8	89	119	0.27	93	135	–44	I	4.8 × 10^–1^	4.9 × 10^–2^	ST	5 × 10^–4^	<1 × 10^–5^	3.8 × 10^–1^
*h*A_l_	*h-*MAA_350_	4.2	1188	>10k	0.40	1520		–47	II^#^	7.0 × 10^2^	2.4 × 10^–2^	ST	2 × 10°	9.4 × 10^–2^	2.2 × 10^–1^
*h*A_s_	*h-*MAA_8_	4.2	120	882	0.24	173		–68	II^#^	7.2 × 10^1^	1.2 × 10^–2^	ST	3 × 10^–1^	4.0 × 10°	5.9 × 10°
*L*SDS	SDS	1.0	89	121	0.11	72	70	–44	I	2.7 × 10^–2^	2.9 × 10^–2^	N	n.a	<1 × 10^–5^	2.0 × 10^–1^

a Macromonomer characteristics as listed in Table 1 and recipe emulsion polymerization according to Table 2; more detailed results for each latex can be found in the SI.

b Subscript “l” denotes a latex prepared with a relatively large and “s” a relatively short macromonomer.

c Added mass of macromonomer or SDS relative to the total amount of added monomer.

d Number-average diameter determined by respectively DLS (standard model CUMULANT, ^$^used model CONTIN), SEM, and TEM.

e *D*_H_ by DLS.

f ζ*-*potential in diluted sodium carbonate solution (ca. 0.01 wt %) at *pH* = 10.

g Visual appearance of latex: type I: liquid-like latex, type II: weakly flocculated latex, type III: gel-like latex,^67^^#^(some) sedimentation in time.

h η_min_ and τ_min_ at γ̇ = 0.001 s^–1^, *η_min_ measured at lowest shear rate within measurement range of rheometer (γ̇ ≈ 0.02 s^–1^), η_max_ at γ̇ = 100 s^–1^, *G*′ < 1 × 10^–5^ Pa and n.a. means too low to be give reliable value.

i Behavior under shear: ST = shear thinning; *N* = Newtonian.

**Figure 3:**
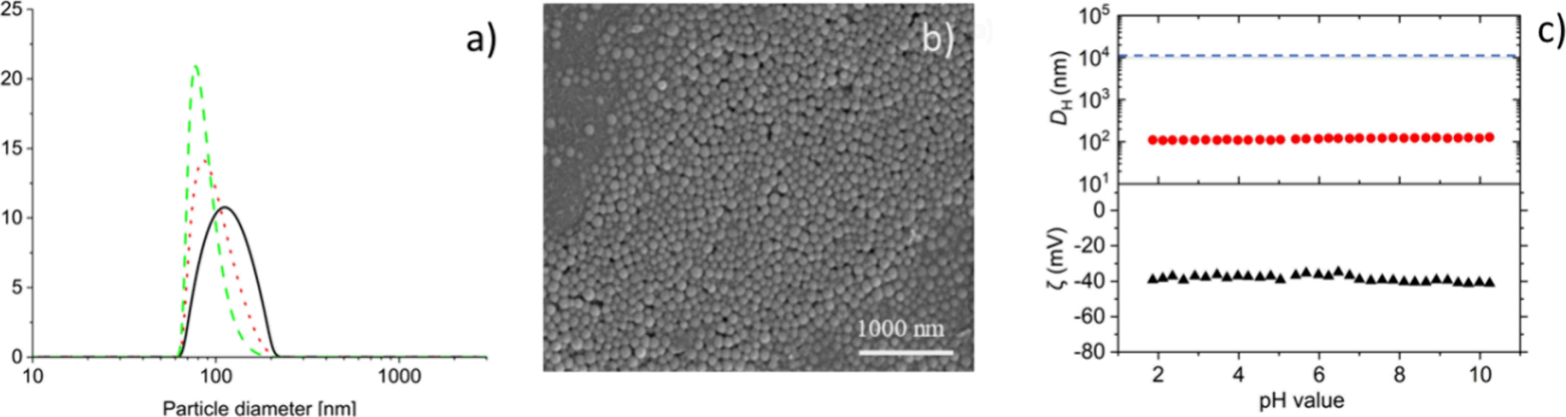
Overview of results DLS and SEM of a type I latex: *b*M, (a) PSD: intensity weighted (−); volume weighted (**..**) and number weighted (--) calculated using the CUMULANT model, (b) SEM image of diluted latex; (c) zeta potential (triangle up solid) and *D*_H_ (circle solid) as a function of the pH of diluted latex, which is adjusted to pH = 10 with 0.1 M NaOH solution and titrated with 0.01 M HCl solution; the horizontal blue line at *D*_H_ = 10^4^ nm (10 μm) indicates the instrument measuring threshold for particle sizes.

For the type I latexes, the PSD is in general narrower, and the hydrodynamic diameters (e.g., *D*_H_ for *b*M ≈ 120 nm) are only slightly larger than the average diameters *(D*_n_, *D*_v_, and *D*_I_). The small increase in *D*_H_ compared to *D*_n_ measured using DLS, SEM, or TEM is due to swelling of carboxylic groups at the surface of the latex particles.^70^ The results for a typical type I latex: *b*M are shown in Figure 3.

Since the *D*_n_ values determined from the SEM and TEM images (*D*_n,SEM_ and *D*_n,TEM_) are comparable to the *D*_n_ calculated from the DLS measurements (*D*_n,DLS_), we will use the *D*_n_ values to compare the particle sizes of the different latexes. When we look at the *D*_n,SEM_ of all latexes, it is clear that these are the smallest in the case of *b*M and *s*L_s_ and are comparable to the particle diameter of the reference SDS latex (*L*SDS) (∼75 nm). *h*A_l_ has the largest particle diameter (∼1200 nm); in this case, a long macromonomer consisting of only MAA was used and the time to form (micelles and) primary particles was long (>8 h) (see Figure 1b), and so finally fewer and therefor larger particles were formed.

**Figure 4:**
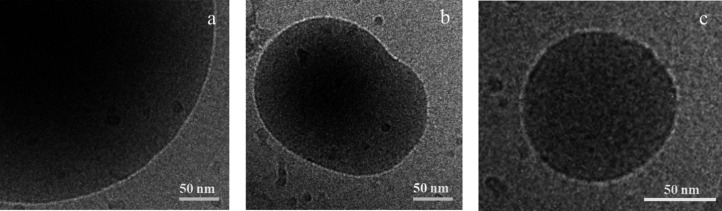
Cryo-TEM images of (a) *s*B_l_; (b) *s*M_l_, and (c) *s*M_s_ with hairy features visible on the surface.

Considering all latexes, we see large differences in the *D*_H_ (in Table 3). *D*_H_ is a measure of the particle diameter in the water phase, including the shell of stabilizing surfactants absorbed or anchored on the particle surface and the hydrophilic chains extended into the water phase. To investigate this shell of surfactant molecules, we measured ζ and *D*_H_ as a function of the pH from basic (*pH* = 10) to acidic (*pH* = 2); for each latex a plot of *D*_H_ as a function of pH is shown in the SI. By reducing the pH, the charge of the anionic (MAA) groups in the surfactant will be neutralized, the shell, if present, will collapse, and the electrosteric stabilization will be reduced. This will result in a decrease (of the absolute value) of ζ and potentially an increase of *D*_H,_ when particles agglomerate into larger flocs. At low pH (*pH* < 4), we see a large decrease of the (absolute) value of ζ for most latexes. Only *b*M shows no influence at all on both ζ and *D*_H_, which is more comparable to *L*SDS. Also, for *s*L_s_, *D*_H_ is constant from pH = 10 to pH = 2.5, although ζ is gradually decreasing. This lack of influence of the pH on *D*_H_ suggests that the surfactants in these three latexes are not extending from the particle surface into the water phase but stay more on the surface.^71^ The influence on *D*_H_ for *s*L_l_, *s*B_l_, *s*M_l_, and *h*A_l_ could not be investigated because *D*_H_ was above or close to the measurement threshold of the instrument. This high *D*_H_ is caused by large chains extending from the surface into the water phase, thus forming a strong internal network. This steric stabilization not only increases the hydrodynamic size of the particles but also increases the low shear viscosity (η_min_) of the latex and results in a shear thinning latex (see below).^71,72^ The presence of these extending chains (or hairy structures) was confirmed by cryo-TEM (Figure 4), and small features interrupting the under focus rings are visible (for higher magnification and more details see the SI, Figure S15).

In general, we can say that latexes with a larger steric component (*s*L_l_, *s*B_l_, *s*M_l_, and *h*A_l_) show a higher *D*_H_ and a lower ζ compared to the latexes stabilized by the shorter macromonomers (*s*L_s_, *s*B_s_, *s*M_s_, and *h*A_s_).

**Figure 5:**
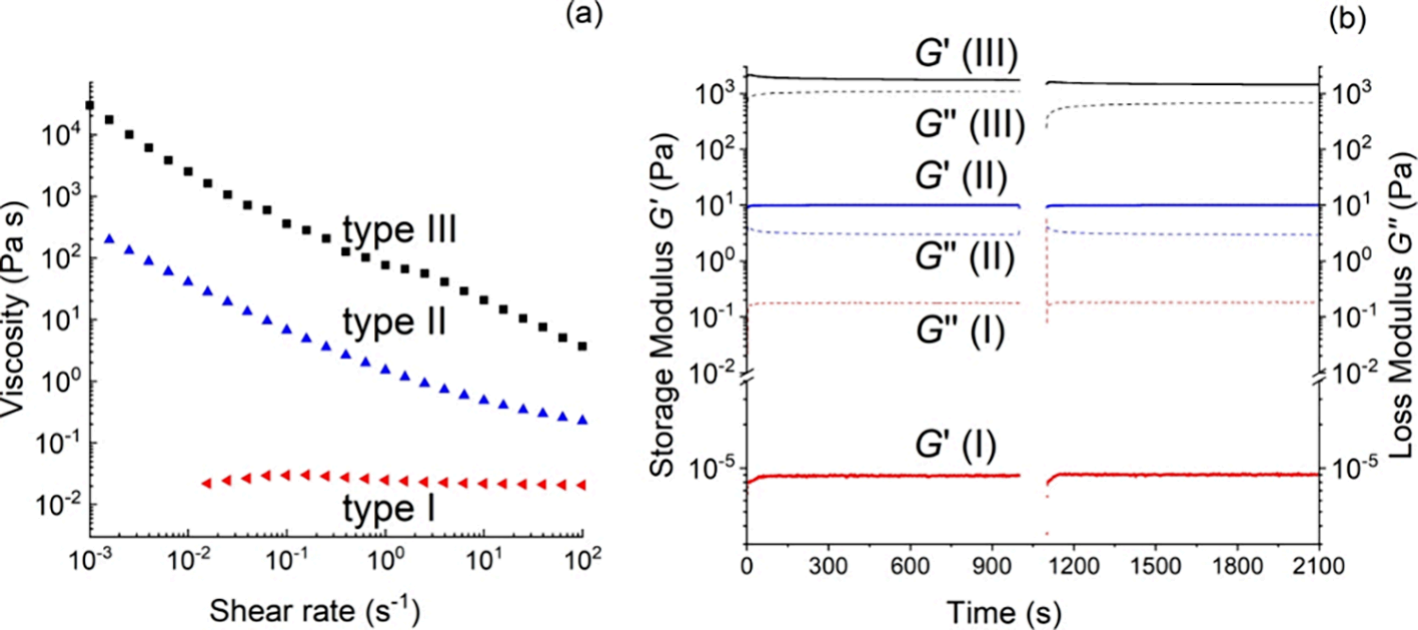
Rheology of p(MMA-*co*-BA) latex according to type: type I: liquid-like, *s*M_s_; type II: viscous latex with reversible flocculation, *s*B_l_; type III, gel-like latex, *s*L_l_; (a) steady state viscosity as a function of shear rate; (b) storage modulus *G*′ (solid line) and loss modulus *G*″ (dotted line) as a function of time.

To get a more quantitative insight into the rheological properties of the prepared latexes, we measured the steady state viscosities as a function of the shear rate (Figure 5a) and performed a dynamic time sweep for a period of 1000 s to probe the mechanical microstructure to determine the dynamic moduli *G*′ and *G*″. To check the recoverability of the structure after such a time sweep, the sample was stirred (using a shear rate of 100 s^–1^ for a duration of 100 s), after which another dynamic time sweep was recorded (Figure 5b). In Figure 5, typical results of the viscosity measurements and the dynamic time sweep experiments are shown for all types of latexes (for details of all latexes, see the SI). Type I latexes show a typical Newtonian behavior: viscosities are independent of the shear rate (Figure 5a) and these latexes show liquid-like behavior (*G*″ ≫ *G*′, Figure 5b). Both type II and III latexes show clear shear thinning behavior, although the viscosities of type III are higher than those of type II over the full range of shear rates; accordingly, the storage and loss moduli of types II and III are also higher than those of type I. *s*L_l_ is the only type III latex. Both *s*L_l_ (type III) and *s*B_l_ (type II) show *G*′ > *G*″, indicating viscoelastic solid-like behavior (see Figure 5b). The other type II latexes, *h*A_l_ and *h*A_s_, also show high values for *G*′, but *G*′ < *G*″, so their behavior is more liquid-like (see the SI for details).

High values of *G*′ and *G*′ > *G*″ for type II and III latexes indicate the presence of strong internal structures, which can be broken up by stirring, but after stirring are immediately restored to the same level (see Figure 5b). These four latexes show clear shear thinning behavior and at high shear rates the viscosities tend to level off, suggesting that the internal structures are no longer broken up by increasing shear rates; the leveling off takes place at relatively high viscosities suggesting that large agglomerates or flocs with strong interactions still exist.^29^ Also, *s*L_s_ and *b*M (both type I) show shear thinning, but at much lower viscosities, and already at shear rates of >0.01 s^–1^, the viscosities level off (η_max_ ≈ 1 × 10^–2^ Pa·s). All these shear thinning latexes show a slope of around −1 in a double-log plot, indicative of plastic behavior; this means that large agglomerates stick to each other and form soft solids with stresses, τ_min_, ranging from ∼20 (for *s*L_l_) to ∼4 × 10^–4^ Pa (for *s*L_s_ and *b*M) at the lowest measured shear rate (γ̇ = 0.001 s^–1^) (see Table 3 and Figure S17). The internal network at rest or a low shear rate is caused by the interaction of charged polymer chains present on the particle surface at the high pH values of the latexes. These anionic charges will repel each other, and the polymers swell and uncoil occupying more volume within the water phase. Additionally, the hydrophobic groups present can form domains along other hydrophobic groups giving a more structured, less liquid-like system.^73^ The other four latexes (*s*B_s_, *s*M_l_, *s*M_s_, *L*SDS) showed very low and almost constant viscosities (η ≈ 1 × 10^–2^ Pa·s) at increasing shear rates (Newtonian) and no (yield) stresses were measurable; this liquid-like behavior was confirmed by the dynamic measurements (*G*” ≫ *G*′; *G*′ ≈ 0).

Next, we characterized the polymers synthesized in the emulsion polymerizations. In Table 4, average molar masses determined by SEC, glass transition temperatures (*T*_g_) determined by DSC and DMA, and water absorption of the bulk polymer measured using DVS are listed (see details per latex in the SI). In general, the values for *T*_g_ measured using DSC were only slightly different from those estimated using the Fox equation^74^ and slightly higher than the *T*_g_ of the reference emulsion polymer (*L*SDS), because of the incorporation of the (higher *T*_g_) macromonomer in the polymer chains. For *s*L_l_, we see a broad glass transition; here, copolymers were formed with different built-in amounts of (macro)monomer, probably caused by diffusion limitations at the end of the polymerization due to the high viscosity of this latex.

**Table 4:** Characteristics of Synthesized Emulsion Polymers^a^

	*T*_g,DSC_	*T*_g,DMA_	*T*_g,Fox_^b^	*G*′_0_^c^	*M*_n,SEC_^d^	*M*_w,SEC_^d^	
latex ^#^	°C	°C	°C	MPa	g mol^–1^	g·mol^–1^	water absorption^e^ (%)
*s*L_l_	10	-f	12	-f	1.1 × 10^5^	5.9 × 10^5^	5.3
*s*L_s_	13	27	8	0.4	1.6 × 10^5^	7.5 × 10^5^	1.6
*s*B_l_	20	32	13	1.1	8.4 × 10^4^	3.7 × 10^5^	2.8
*s*B_s_	12	12	8	0.6	8.9 × 10^4^	5.1 × 10^5^	12.0
*s*M_l_	14	24	13	0.6	6.4 × 10^4^	2.0 × 10^5^	4.1
*s*M_s_	8	21	14	0.8	6.5 × 10^4^	4.7 × 10^5^	7.1
*b*M	7	22	13	0.6	8.5 × 10^4^	4.9 × 10^5^	1.7
*h*A_l_	17	48	13	0.5	9.9 × 10^4^	5.4 × 10^5^	2.6
*h*A_s_	7	20	10	0.9	8.4 × 10^4^	4.2 × 10^5^	1.4
*L*SDS	8	30	8	0.2	7.8 × 10^4^	3.8 × 10^5^	6.1

a Latex properties listed in Table 3.

b *T*_g_ calculated with the Fox equation,^74^ (see eq 1 in SI) using measured *T*_g_s of macromonomers listed in Table 1.

c Storage modulus at rubbery plateau.

d Number-average (*M*_n_) and weight-average (*M*_w_) molar mass determined by SEC (using THF) relative to polystyrene standards.

e Water absorption *=* (*M*_95_ – *M*_0_)*/M*_0;_*M*_95_ and *M*_0_ are sample masses measured at 95% and 0.5% RH, respectively.

f No adequate film formation.

The SEC results in Table 4 show very broad molar mass distributions irrespective of the used stabilizer. It is known that although the macromonomers can function as chain transfer agents, they will undergo complex copolymerization^49^ during the emulsion polymerization and can be buried inside the polymer particles. The resulting polymers are characterized (using DMA) by a relatively constant rubbery plateau (*G*_0_) between ∼0.5 and ∼1 MPa as is clear from Table 4.

**Figure 6:**
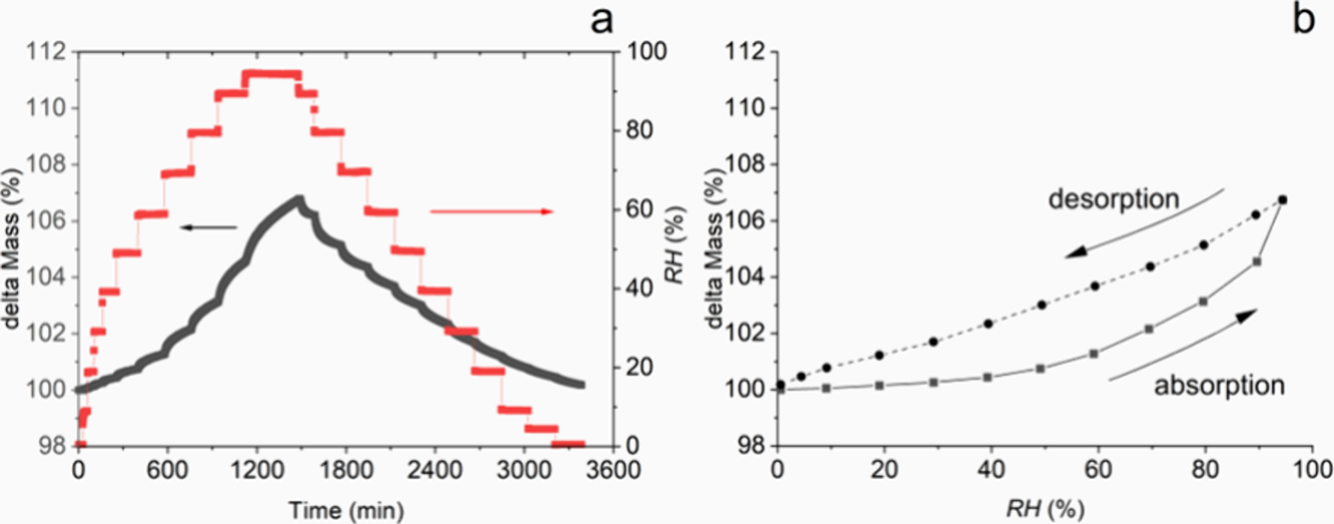
(a) Water vapor sorption kinetics for *L*SDS as a function of the relative humidity (RH) in time. The black line is the change in sample mass during the first sorption and subsequently desorption. The red line is the RH in the cell; (b) water vapor sorption isotherm for LSDS as a function of RH, lower (solid) curve corresponds to absorption, and upper (dashed) curve to desorption.

Finally, dynamic water vapor sorption analyses were performed for all 10 polymers. The equilibrium relative humidity (RH) in the cell was varied from 0.5% (i.e., the dry state) to 95% (i.e., the wet state) and back to 0.5% with steps of 5–10%. The change in mass of the polymer at each RH was measured constantly, and after reaching equilibrium (d*m*/d*t* < 0.005 wt %/min), the RH was increased (sorption) or decreased (desorption) to the next value. The results for a typical sorption–desorption experiment are shown in Figure 6 for the reference latex *L*SDS.

**Figure 7:**
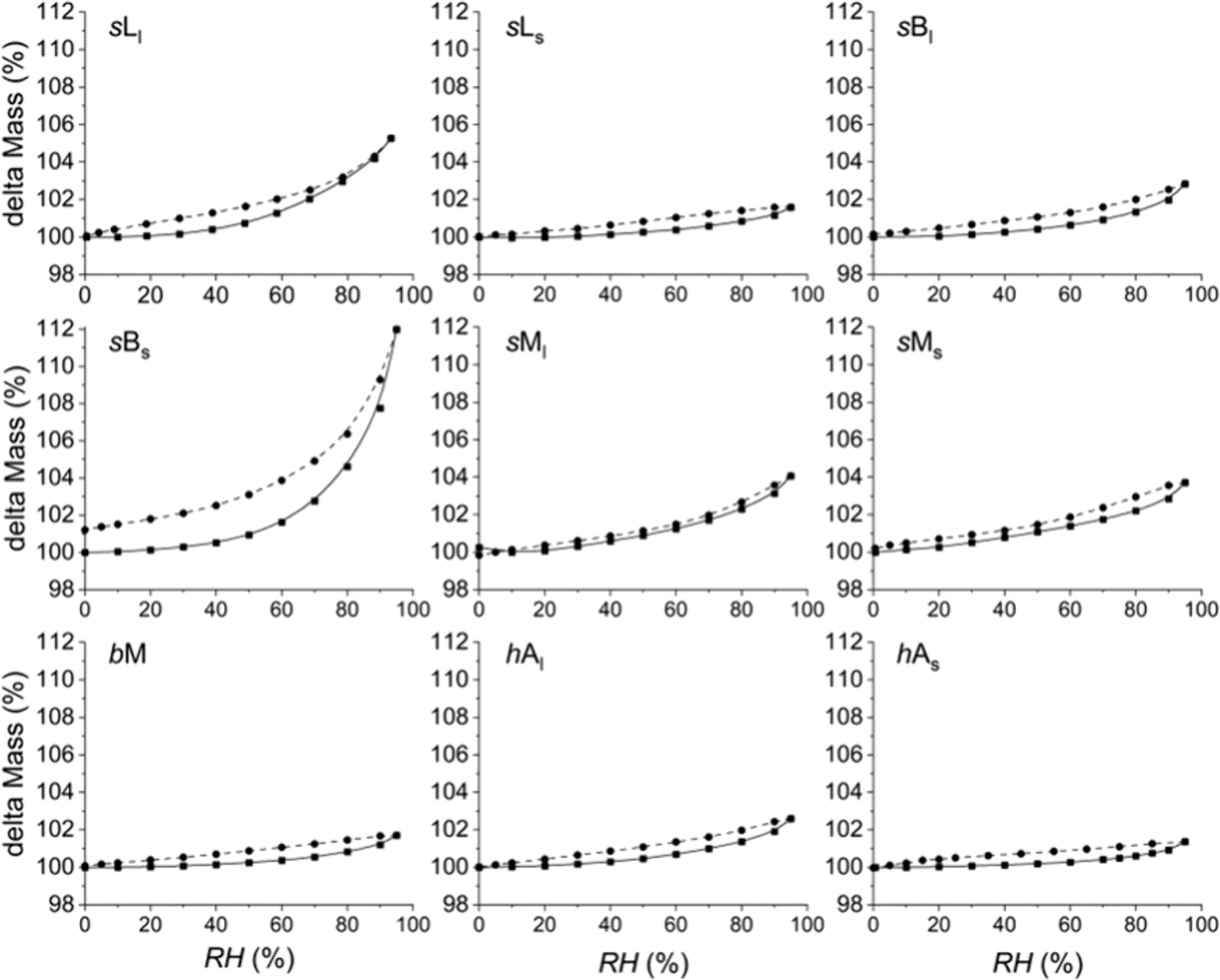
Water sorption isotherms for the different emulsion polymers p(MMA-*co*-BA) as a function of the partial water vapor pressure or relative humidity (RH) over a range from 0 to 95%. The lower (solid) curve indicates absorption, and the upper (dashed) curve desorption.

Typically, we see an increase of the mass when the RH increases and a decrease of the mass when the RH decreases. The gap between the sorption and desorption isotherms in Figure 6b is indicative for bulk sorption/desorption.^75^ The isotherms of all other emulsion polymers are plotted in Figure 7 (see SI for more details), and all polymers showed bulk sorption/desorption behavior, indicating that no micro- or mesopores are present in the material. This behavior is typical for hydrophobic materials containing polar groups.^75−77^ Except for *s*B_s_, all emulsion polymers containing macromonomer have a water sorption between 1 and 5 wt %, which is lower than that of the reference polymer (*L*SDS), despite the use of the very hydrophilic MAA comonomers (0.025 mol −COO^–^ ≫ 0.002 mol −OSO_3_^–^).The high water sorption for *s*B_s_ occurs at high RH (>40%) and is probably the result of hydroplasticization.^78^

### Structure and Properties of Polymer Coatings

Thin films were cast directly from latex onto a glass substrate and dried overnight. Four preparation methods were investigated: latexes were just dried at either 20 (prep A) or 40 °C (prep B); latexes were dried at 20 °C and annealed at 60 °C for 5 days (prep C) or dried at 40 °C and annealed at 60 °C for 5 days (prep D). Only the results of prep A and/or D, showing the extremes, will be discussed in the remainder of the paper (see the SI for results of all preparations). For adhesion and gloss measurements, latex films were cast onto an aluminum substrate and dried at 20 °C overnight (prep A only). The surfaces of all prepared films were examined visually and imaged using AFM and SEM. Furthermore, physical properties such as roughness, hardness, cross hatch adhesion, and gloss were determined. Additionally, the contact angles of the coatings and the interfacial tensions of water droplets on the surface of the coatings were measured using goniometry and the diffusion coefficients and permeabilities for water vapor were determined by DVS. The physical properties of the films are summarized in Table 5.

**Table 5:** Physical Properties of Polymer Films^a^ Prepared with Different Preparation Methods (Prep A and/or Prep D)^b^

				AFM		König hardness^d^				goniometry^g^			DVS^h^	
				*R*_a_^c^			gloss^e^			contact		γ_lg_	*D*_dif_	*P*
	*T*_g,DSC_	visual appearance		nm		s	GU	%	adhesion^f^	°		mN·m^–1^	cm^2^·s^–1^	g·h^–1^·m^–2^·mm
latex	°C	A	D	A	D	A	A		A	A	D	A	A	A
*s*L_l_	10	opaque	hazy	11	10	-i	59	37	-i	71	88	71.8	-i	-i
*s*L_s_	13	clear	clear	4	2	38^i^	99	61	5B	61	70	72.1	1.4 × 10^–8^	2
*s*B_l_	20	clear	clear	10	8	45	97	60	4B	48	60	69.5	4.8 × 10^–8^	8
*s*B_s_	12	clear	clear	6	3	14	89	55	4B	36	21	41.4	1.1 × 10^–7^	17
*s*M_l_	14	hazy	clear	17	7	20	87	54	4B	67	<10	54.4	6.6 × 10^–8^	5
*s*M_s_	8	clear	clear	5	6	13	96	59	4B	52	55	66.9	1.0 × 10^–7^	14
*b*M	7	clear	clear	2	1	13	96	60	2B	69	81	72.0	8.6 × 10^–8^	8
*h*A_l_	17	opaque	opaque	144	63	-i	22	14	-i	65	67	66.6	-i	-i
*h*A_s_	7	clear	clear	8	6	17	54	34	1B	21	81	62.2	1.5 × 10^–7^	16
*L*SDS	8	clear	clear	12	9	26	94	58	4B	46	60	71.6	5.7 × 10^–8^	6

a Latex properties listed in Table 3.

b Prep A (A): film dried at 20 °C, prep D (D): film dried at 40 °C and annealed at 60 °C.

c *R*_a_ is roughness of the surface at an evaluation length of 5 μm measured using AFM.

d Oscillating time measured according to ASTM 4356.^79^

e Gloss values (in gloss units (GU)) and % reflection at 85°.

f Cross hatch adhesion measured according to ASTM 3359^80^ scale from 5B (no damage) to 0B (fully detached).

g Water contact angle measured via sessile drop method, γ_lg_ is the water–air interfacial tension of a water droplet from the coating surface measured via pendant drop method,^81^ and γ_lg_ of water in air is 72.1 mN·m^–1^.

h Diffusion coefficient (*D*_dif_) and permeability (*P*) of water vapor through a thick film using a Payne cell.^82^

i Not measured.

**Figure 8:**
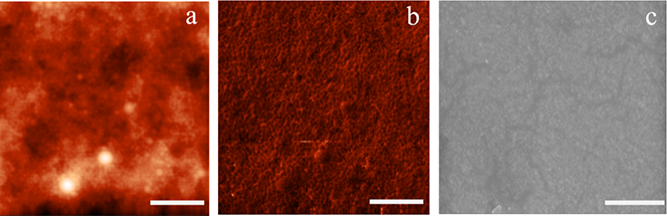
AFM and SEM images of *s*L_s_; (a) AFM height and (b) phase image of a film (prep A); (c) SEM image of a dried film (prep A), coated with 10 nm gold before imaging, bar 1 μm.

Most of the coatings prepared at room temperature (prep A) were clear coatings as expected from the *T*_g_ for these MMA-BA copolymers; interdiffusion of polymer chains to coalesce the latex particles during film formation can only occur above the *T*_g_ of the polymers.^69,82−84^ As an example for a clear coating, the AFM and SEM images of *s*L_s_ are shown in Figure 8. From the AFM images (Figure 8a,b), it can be seen that the surface is rather homogeneous, only some larger (flattened) particles (height ≈ 20 nm) are visible, and the average roughness (*R*_a_) of the surface is only 4 nm; an *R*_a_ ≤ 4 nm is considered smooth.^85^ Additionally, SEM (Figure 8c) of the same film showed a smooth surface.

**Figure 9:**
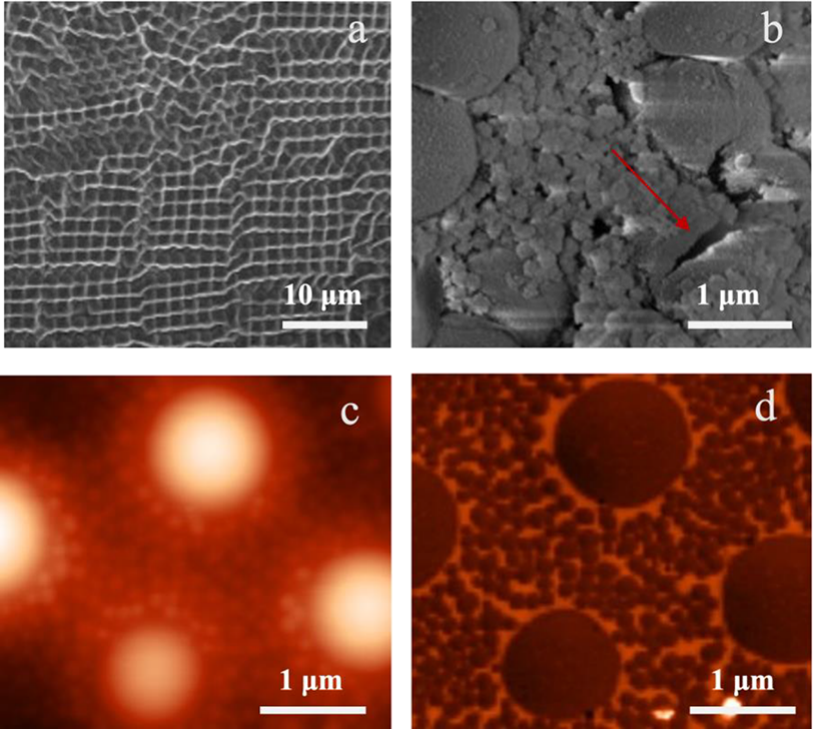
AFM and SEM images of *s*M_l_; (a) SEM image of an area showing closely packed large particles; (b) SEM image showing an area with ∼1 μm particles surrounded by ∼120 nm polymer particles, the arrow points toward a broken particle; (c) AFM height and (d) phase image of a film (prep A). SEM images were prepared from a diluted latex, dried at 20 °C, and coated with 10 nm gold before imaging.

When we analyze one of the hazy films, *e.g., s*M_l_ (prep A), using SEM (Figure 9a), we see areas showing closely packed large particles (>1 μm). Zooming in at other areas (Figure 9b), we also see large particles but now surrounded by many small particles (∼120 nm); this is also observed in the DLS data measured directly after polymerization (see SI), implying that the large particles were formed during polymerization. The presence of smaller particles is desirable to improve the film formation by increasing the capillary forces needed for coalescence.^86,87^ These large particles surrounded by smaller ones are also observed using AFM (Figure 9c,d) and in AFM and SEM for some of the other coatings (*s*L_l_, *s*M_s_, and *h*A_l_); all these coatings contain a high *T*_*g*_ macromonomer (see SI for AFM images of the other films). When considering the SEM image in more detail, we observe that these large particles seem to be hollow (see some broken particles in the SEM image in Figure 9b). A similar observation was previously described in the literature for core–shell particles with different *T*_g_ values in the core and shell using (meth)acrylic acid as a comonomer and was ascribed to the soft BA/MMA core flowing out of the hard MMA shell.^85^ Something similar could also have happened in our case and if these large particles contain water or air; this inclusion can result in hazy or opaque films,^86,87^ which is also observed for films from *s*L_l_, *s*M_l_, and *h*A_l_. To improve the appearance of the hazy films (*i.e., s*L_l_, *s*M_l_, and *h*A_l_), the films were annealed at 60 °C (prep D) and even 100 °C for 5 days. This resulted in a clear film for *s*M_l_ but not for *s*L_l_ and *h*A_l_; therefore, these two coatings were not investigated further.

Using AFM, we also analyzed the roughness of the films prepared by using different drying methods. We observed that drying the films at 40 °C (prep B) instead of 20 °C (prep A) does not have a large influence on the roughness; due to the decreased drying time at higher temperatures, the particles have less time to coalesce. Annealing of both films at 60 °C (preps C and D) decreased the roughness significantly (see Table 5). All clear coatings appear to have a roughness (*R*_a_) that is lower than that of the reference coating (*L*SDS).

We also measured the gloss, hardness, and adhesion of the coatings using prep A and compared these results with those of the reference coating *L*SDS (Table 5). The very low roughness of all coatings results in high gloss (i.e., gloss values >80 GU^88^); all films except for *h*A_s_ show gloss values of >87 GU. Cross hatch adhesion of the different coatings, except those of *b*M and *h*A_s_, was better than that of *L*SDS. For the films of *s*L_s_, *s*B_l_, and *b*M, the measured hardness values are higher than for *L*SDS. The coating with the highest *T*_g_ (*s*B_l_), which is also just above the measuring temperature (*T* ≈ 19 °C), appears to be also the hardest, and we see that the surfactants with the shortest chains (*s*B_s,_*s*M_s_, *b*M, *h*A_s_) give the most damping (less oscillations) and thus appear to be the softest.

The presence of hydrophilic groups, such as MAA, at the surface of coatings is expected to have a large influence on the surface properties, so we will also discuss some relevant important physical properties, of the coatings: the water contact angle and interfacial tension of the surface, the diffusion coefficient, and the permeability of the film for water vapor. The water contact angle and the interfacial tension of water droplets on the surface (γ_lg_) were measured using goniometry and the results are also listed in Table 5. Since all contact angles are below 90°, we can conclude that all coatings are hydrophilic. The hydrophilicity of the films decreases (increase of contact angle) in most cases when the films are dried at a higher temperature and after annealing. In most cases, the coatings are more hydrophobic than the reference *L*SDS coating, which was not immediately expected, especially because the number of acid groups in MAA (0.025 mol) in the reaction is 10 times higher than the number of hydrophilic sulfate groups of SDS (0.002 mol). The contact angles of coatings prepared using the longer macromonomers (*s*L_l_, *s*B_l_, *s*M_l_, and *h*A_l_) are higher than those of the coatings prepared using the shorter chain analogues (*s*L_s_, *s*B_s_, *s*M_s_, and *h*A_s_). The short macromonomers, which lead to better electrostatic stabilization of the latex particles (higher ζ potential), are located more at the surface of the particles and after drying also make the coating surface more hydrophilic. To determine whether the initial macromonomers are covalently bound to the polymer particles, we measured the interfacial tension (γ_lg_) of a water droplet originating from the coating surface. For *s*L_l_, *s*L_s_, and *b*M, we see no change in the interfacial tension compared to pure water (γ_lg_ for water–air is 72.1 mN·m^–1^), so no surface-active molecules were dissolved from the coating surface. The coatings prepared from *s*B_s_, *s*M_l_, *s*M_s_, *h*A_l_, and *h*A_s_ gave a decrease of the γ_lg_, and in the case of *s*B_s_, *s*M_l_, and *s*M_s_, the water droplet also became white, indicating that molecules from the surface dissolved into the water droplet. A further indication that this is indeed the case is the observation that the supernatants of these latexes also contained water-soluble polymers (see SI, Figure S22 for SEC analyses of these supernatants).

We also measured whether the presence of MAA groups has an influence on the diffusion coefficient (*D*_Dif_) and the permeability (*P*) of the coatings for water vapor. The thickness of the films was around 500 μm. We measured the mass change as a function of time using a Payne cell with DVS for all coatings (see Table 5 for *D*_Dif_ and *P* values and see the SI for the used equations and absorption curve). From the absorption curves, it could be concluded that the sorption of water vapor through the films is Fickian. All diffusion coefficients are between 1.5 × 10^–7^ and 1.4 × 10^–8^ cm^2^·s^–1^ and the permeabilities between 2 and 17 g·h^–1^·m^–2^·mm. The coatings with the lowest contact angles (highest hydrophilicity) show a slightly higher *D*_Dif_ and *P* for water vapor than the less hydrophilic coatings. These differences are comparable to those of other polymers with small differences in chemical nature (like polarity) but minor compared to those for polymers showing more structural differences (like crystallinity, chain stiffness, and cross-linking).^82,89−91^ The presence of MAA groups on the surface can increase the initial absorption of water at high RH (RH > 60%) and, through the hydroplasticization effect, increase the permeability.^78,92,93^ At lower humidity (RH < 50%), the permeability is more controlled by temperature and concentration differences over the two sides of the film, and the absorbed water molecules in the hydrophobic polymer will be more clustered and not contribute to the permeability.^78,91,92,94^

## Conclusions

In this work, we prepared coatings from latexes synthesized with BA and MMA stabilized with methacrylic acid (MAA) containing macromonomers having different architectures and compositions. Although we kept the overall composition of the monomers in the emulsion constant and used equal amounts of MAA in each emulsion polymerization, we obtained latexes and coatings with rather diverse properties, which allow tuning for particular applications. For example, very low water permeabilities are obtained for *s*L_s_, whereas a higher permeability is obtained for *s*B_s_. In construction applications, the former is required for corrosion protection,^21^ whereas the latter is required for wood protection. We have also seen that coatings of different hardnesses can be obtained, which may also be important for certain cell-surface interactions. Small differences in the macromonomer microstructure were also found to affect the colloidal and rheological properties of the polymer latexes. The relatively high solids contents in these studies may not always be directly relevant for biomedical applications, but what causes the observed differences are the differences in “hairiness” of the particles, and this is of obvious importance in diagnostic and therapeutic application of polymer particles as a hairy layer around the particles will affect the interactions of the particles with their environment and with other molecules. For the application of a coating, the viscosity of the latex during and after application is also of obvious importance. Some of our latexes show shear thinning behavior at different viscosity levels, and others are more Newtonian. For example, *s*L_s_ is a shear thinning latex with a small yield stress, which resulted in a high gloss, clear coating, which was hard and smooth, proved to have good adhesion on aluminum, and showed a low water permeability. Finally, we conclude that only relatively minor changes in the microstructure of reactive polymeric surfactants may lead to a large range of particle and film properties and that it is worthwhile to investigate these small changes in any system of interest.

## Supporting Information Available

Detailed experimental details of the characterization techniques, summaries of all characterization results for each latex/coating, comparisons of DLS data, additional AFM results, additional SEM and cryo-TEM images, additional rheological data, contact angles, comparison of DVS sorption plots, additional kinetic plots of the emulsion polymerization, and SEC data of the supernatants of the produced latexes (PDF)

## References

[ref1] Khor S. Y.; Quinn J. F.; Whittaker M. R.; Truong N. P.; Davis T. P. Controlling Nanomaterial Size and Shape for Biomedical Applications via Polymerization-Induced Self-Assembly. Macromol. Rapid Commun. 2019, 40 (2), 1800438 10.1002/marc.201800438. 30091816

[ref2] Kupikowska-Stobba B.; Kasprzak M. Fabrication of Nanoparticles for Bone Regeneration: New Insight into Applications of Nanoemulsion Technology. J. Mater. Chem. B 2021, 9 (26), 5221–5244. 10.1039/D1TB00559F. 34142690

[ref3] Dalei G.; Das S. Polyacrylic Acid-Based Drug Delivery Systems: A Comprehensive Review on the State-of-Art. J. Drug Deliv Sci. Technol. 2022, 78, 103988 10.1016/j.jddst.2022.103988.

[ref4] Alvi M.; Yaqoob A.; Rehman K.; Shoaib S. M.; Akash M. S. H. PLGA-Based Nanoparticles for the Treatment of Cancer: Current Strategies and Perspectives. AAPS Open 2022, 8 (1), 12 10.1186/s41120-022-00060-7.

[ref5] Elaissari A. Magnetic Colloids: Preparation and Biomedical Applications. e-Polym. 2005, 5, 1 10.1515/epoly.2005.5.1.296.

[ref6] Chiu H.-C.; Chern C.-S.; Lee C.-K.; Chang H.-F. Synthesis and Characterization of Amphiphilic Poly (Ethylene Glycol) Graft Copolymers and Their Potential Application as Drug Carriers. Polymer (Guildf) 1998, 39 (8), 1609–1616. 10.1016/S0032-3861(97)00436-9.

[ref7] Zhang W. J.; Hong C. Y.; Pan C. Y. Polymerization-Induced Self-Assembly of Functionalized Block Copolymer Nanoparticles and Their Application in Drug Delivery. Macromol. Rapid Commun. 2019, 40 (2), 1800279 10.1002/marc.201800279. 29968349

[ref8] Velev O. D.; Kaler E. W. In Situ Assembly of Colloidal Particles into Miniaturized Biosensors. Langmuir 1999, 15 (11), 3693–3698. 10.1021/la981729c.

[ref9] Tan C. J.; Chua H. G.; Ker K. H.; Tong Y. W. Preparation of Bovine Serum Albumin Surface-Imprinted Submicrometer Particles with Magnetic Susceptibility through Core-Shell Miniemulsion Polymerization. Anal. Chem. 2008, 80 (3), 683–692. 10.1021/ac701824u. 18181645

[ref10] Qu Z.; Xu H.; Gu H. Synthesis and Biomedical Applications of Poly((Meth)Acrylic Acid) Brushes. ACS Appl. Mater. Interfaces 2015, 7 (27), 14537–14551. 10.1021/acsami.5b02912. 26067846

[ref11] Wang L.; Wong Y. C.; Correira J. M.; Wancura M.; Geiger C. J.; Webster S. S.; Touhami A.; Butler B. J.; O’Toole G. A.; Langford R. M.; Brown K. A.; Dortdivanlioglu B.; Webb L.; Cosgriff-Hernandez E.; Gordon V. D. The Accumulation and Growth of Pseudomonas Aeruginosa on Surfaces Is Modulated by Surface Mechanics via Cyclic-Di-GMP Signaling. NPJ Biofilms Microbiomes 2023, 9 (1), 77 10.1038/s41522-023-00436-x. 37816780 PMC10564899

[ref12] Techakasikornpanich M.; Jangpatarapongsa K.; Polpanich D.; Elaissari A. Impact of Polymeric Films and Hydrogels: Physical Characteristics on Bacterial Growth. Polym. Adv. Technol. 2024, 35 (2), 1–19. 10.1002/pat.6311.

[ref13] Álvarez-Paino M.; Muñoz-Bonilla A.; López-Fabal F.; Gómez-Garcés J. L.; Heuts J. P. A.; Fernández-García M. Functional Surfaces Obtained from Emulsion Polymerization Using Antimicrobial Glycosylated Block Copolymers as Surfactants. Polym. Chem. 2015, 6 (34), 6171–6181. 10.1039/C5PY00776C.

[ref14] Elaissari A.; Sauzedde F.; Montagne F.; Pichot C. Colloidal Polymers: Synthesis and Characterization, 1st ed.; Elaissari A., Ed.; Marcel Dekker, Inc.: NewYork, 2003.

[ref15] Aguirre M.; Ballard N.; Gonzalez E.; Hamzehlou S.; Sardon H.; Calderon M.; Paulis M.; Tomovska R.; Dupin D.; Bean R. H.; Long T. E.; Leiza J. R.; Asua J. M. Polymer Colloids: Current Challenges, Emerging Applications, and New Developments. Macromolecules 2023, 56 (7), 2579–2607. 10.1021/acs.macromol.3c00108. 37066026 PMC10101531

[ref16] Shahzadi P.; Majeed M. A.; Ibrahim S.; Asif S.; Kalsoom R.; Hussain I. Polymeric Coating Doped with Nanomaterials for Functional Impact on Different Substrates. Sci. Rep. 2024, 14, 1 10.1038/s41598-023-50462-0. 38182627 PMC10770307

[ref17] Baek S. S.; Jang S. J.; Hwang S. H. Preparation and Adhesion Performance of Transparent Acrylic Pressure Sensitive Adhesives: Effects of Substituent Structure of Acrylate Monomer. Int. J. Adhes Adhes 2016, 64, 72–77. 10.1016/j.ijadhadh.2015.10.005.

[ref18] Wang T.; Canetta E.; Weerakkody T. G.; Keddie J. L.; Rivas U. PH Dependence of the Properties of Waterborne Pressure-Sensitive Adhesives Containing Acrylic Acid. ACS Appl. Mater. Interfaces 2009, 1 (3), 631–639. 10.1021/am800179y. 20355985

[ref19] Lee S. W.; Park J. W.; Kwon Y. E.; Kim S.; Kim H. J.; Kim E. A.; Woo H. S.; Swiderska J. Optical Properties and UV-Curing Behaviors of Optically Clear Semi-Interpenetrated Structured Acrylic Pressure Sensitive Adhesives. Int. J. Adhes Adhes 2012, 38, 5–10. 10.1016/j.ijadhadh.2012.04.002.

[ref20] Hýsek Š.; Fidan H.; Pánek M.; Böhm M.; Trgala K. Water Permeability of Exterior Wood Coatings: Waterborne Acrylate Dispersions for Windows. Journal of Green Building 2018, 13 (3), 1–16. 10.3992/1943-4618.13.3.1.

[ref21] Chen F.; Liu P. Conducting Polyaniline Nanoparticles and Their Dispersion for Waterborne Corrosion Protection Coatings. ACS Appl. Mater. Interfaces 2011, 3 (7), 2694–2702. 10.1021/am200488m. 21699229

[ref22] Huybrechts J.; Bruylants P.; Kirshenbaum K.; Vrana J.; Snuparek J. New Applications of Catalytic Chain Transfer Polymerization to Waterborne Binders for Automotive Paint Systems. Prog. Org. Coat. 2002, 45 (2), 173–183. 10.1016/S0300-9440(02)00042-5.

[ref23] Jiao C.; Sun L.; Shao Q.; Song J.; Hu Q.; Naik N.; Guo Z. Advances in Waterborne Acrylic Resins: Synthesis Principle, Modification Strategies, and Their Applications. ACS Omega 2021, 6 (4), 2443–2449. 10.1021/acsomega.0c05593. 33553862 PMC7859933

[ref24] Maan A. M. C.; Hofman A. H.; de Vos W. M.; Kamperman M. Recent Developments and Practical Feasibility of Polymer-Based Antifouling Coatings. Adv. Funct. Mater. 2020, 30 (32), 2000936 10.1002/adfm.202000936.

[ref25] Muñoz-Bonilla A.; Van Herk A. M.; Heuts J. P. A. Preparation of Hairy Particles and Antifouling Films Using Brush-Type Amphiphilic Block Copolymer Surfactants in Emulsion Polymerization. Macromolecules 2010, 43 (6), 2721–2731. 10.1021/ma9027257.

[ref26] Mori H.; Müller A. H. E. New Polymeric Architectures with (Meth)Acrylic Acid Segments. Prog. Polym. Sci. 2003, 28 (10), 1403–1439. 10.1016/S0079-6700(03)00076-5.

[ref27] Chow R. S.; Takamura K. Effects of Surface Roughness (Hairiness) of Latex Particles on Their Electrokinetic Potentials. J. Colloid Interface Sci. 1988, 125 (1), 226–236. 10.1016/0021-9797(88)90071-9.

[ref28] Munoz-Bonilla A.; Ali S. I.; Del Campo A.; Fernández-García M.; van Herk A. M.; Heuts J. P. A. Block Copolymer Surfactants in Emulsion Polymerization: Influence of the Miscibility of the Hydrophobic Block on Kinetics, Particle Morphology, and Film Formation. Macromolecules 2011, 44 (11), 4282–4290. 10.1021/ma200626p.

[ref29] Russel W. B.; Saville D. A.; Showalter W. R. Colloidal Dispersions, 1st ed.; Batchelor G. K., Ed.; Cambridge University Press: Cambridge, 1991.

[ref30] Raffa P.; Wever D. A. Z.; Picchioni F.; Broekhuis A. A. Polymeric Surfactants: Synthesis, Properties, and Links to Applications. Chem. Rev. 2015, 115 (16), 8504–8563. 10.1021/cr500129h. 26182291

[ref31] Aramendia E.; Mallégol J.; Jeynes C.; Barandiaran M. J.; Keddie J. L.; Asua J. M. Distribution of Surfactants near Acrylic Latex Film Surfaces: A Comparison of Conventional and Reactive Surfactants (Surfmers). Langmuir 2003, 19 (8), 3212–3221. 10.1021/la0267950.

[ref32] Guyot A.; Tauer K. Reactive Surfactants in Emulsion Polymerization. Adv. Polym. Sci. 1994, 111, 43–65. 10.1007/BFb0024126.

[ref33] Guyot A. Advances in Reactive Surfactants. Adv. Colloid Interface Sci. 2004, 108–109, 3–22. 10.1016/j.cis.2003.10.009. 15072924

[ref34] Tauer K.; Zimmermann A.; Schlaad H. New Reactive Block Copolymers as Stabilizers in Emulsion Polymerization. Macromol. Chem. Phys. 2002, 203 (2), 319–327. 10.1002/1521-3935(20020101)203:2<319::AID-MACP319>3.0.CO;2-7.

[ref35] Lee W. H.; Booth J. R.; Bon S. A. F. On Particle Size Distributions in Catalytic Chain Transfer Emulsion Polymerization: Chain-Extension and the Use of Derived Macromonomers as Reactive Surfactants in Emulsion Polymerization. Biomacromolecules 2020, 21 (11), 4599–4614. 10.1021/acs.biomac.0c00766. 32683868

[ref36] Schoonbrood H. A. S.; Asua J. M. Reactive Surfactants in Heterophase Polymerization. 9. Optimum Surfmer Behavior in Emulsion Polymerization. Macromolecules 1997, 30 (20), 6034–6041. 10.1021/ma9701494.

[ref37] Lacroix-Desmazes P.; Guyot A. Reactive Surfactants in Heterophase Polymerization. Part XXII—Incorporation of Macromonomers Used as Stabilizers in Styrene Dispersion Polymerization. Polym. Adv. Technol. 1997, 8 (10), 608–615. 10.1002/(SICI)1099-1581(199710)8:10<608::AID-PAT715>3.0.CO;2-#.

[ref38] Márquez I.; Paredes N.; Alarcia F.; Velasco J. I. Influence of Polymerizable Surfactants on the Adhesion Performance and Water Resistance of Water-Based Acrylic Pressure-Sensitive Adhesives (PSAs). J. Adhes Sci. Technol. 2023, 37 (11), 1770–1788. 10.1080/01694243.2022.2095152.

[ref39] Asua J. M.; Schoonbrood H. A. S. Reactive Surfactants in Heterophase Polymerization. Acta Polym. 1998, 49 (12), 671–686. 10.1002/(SICI)1521-4044(199812)49:12<671::AID-APOL671>3.0.CO;2-L.

[ref40] Guyot A.; Tauer K.; Asua J. M.; Van Es S.; Gauthier C.; Hellgren A. C.; Sherrington D. C.; Montoya-Goni A.; Sjoberg M.; Sindt O.; Vidal F.; Unzue M.; Schoonbrood H.; Shipper E.; Lacroix-Desmazes P. Reactive Surfactants in Heterophase Polymerization. Acta Polym. 1999, 50 (2–3), 57–66. 10.1002/(SICI)1521-4044(19990201)50:2/3<57::AID-APOL57>3.0.CO;2-Y.

[ref41] Mestach D. Reactive Surfactants for Commercial Polymer Dispersions. In Aqueous Polymer Dispersions; Tauer K., Ed.; Progress in Colloid and Polymer Science; Springer Berlin Heidelberg, 2004; Vol. 124, pp 37–41.

[ref42] Chen L.; Yan L.; Li Q.; Wang C.; Chen S. Controllable Synthesis of New Polymerizable Macrosurfactants via CCTP and RAFT Techniques and Investigation of Their Performance in Emulsion Polymerization. Langmuir 2010, 26 (3), 1724–1733. 10.1021/la9037809. 19928970

[ref43] Debuigne A.; Poli R.; Jérôme C.; Jérôme R.; Detrembleur C. Overview of Cobalt-Mediated Radical Polymerization: Roots, State of the Art and Future Prospects. Prog. Polym. Sci. 2009, 34 (3), 211–239. 10.1016/j.progpolymsci.2008.11.003.

[ref44] Shegiwal A.; Wemyss A. M.; Liarou E.; Town J.; Patias G.; Atkins C. J.; Marathianos A.; Lester D. W.; Efstathiou S.; Haddleton D. M. Polymerisable Surfactants for Polymethacrylates Using Catalytic Chain Transfer Polymerisation (CCTP) Combined with Sulfur Free-RAFT in Emulsion Polymerisation. Eur. Polym. J. 2020, 125, 109491 10.1016/j.eurpolymj.2020.109491.

[ref45] Karmilova L. V.; Ponomarev G. V.; Smirnov B. R.; Bel’govskii I. M. Metalloporphyrins as Catalysts of Chain Transfer in Radical Polymerisation and Stereoselective Oxidation. Russ. Chem. Rev. 1984, 53 (2), 132–235. 10.1070/RC1984v053n02ABEH003032.

[ref46] Davis T. P.; Haddleton D. M.; Richards S. N. Controlled Polymerization of Acrylates and Methacrylates1. Journal of Macromolecular Science, Part C 1994, 34 (2), 243–324. 10.1080/15321799408009636.

[ref47] Davis T. P.; Kukulj D.; Haddleton D. M.; Maloney D. R. Cobalt-Mediated Free-Radical Polymerization of Acrylic Monomers. Trends Polym. Sci. 1995, 3 (11), 365–373.

[ref48] Gridnev A. A. 25th Anniversary of Catalytic Chain Transfer. J. Polym. Sci. A Polym. Chem. 2000, 38 (10), 1753–1766. 10.1002/(SICI)1099-0518(20000515)38:10<1753::AID-POLA600>3.0.CO;2-O.

[ref49] Gridnev A. A.; Ittel S. D. Catalytic Chain Transfer in Free-Radical Polymerizations. Chem. Rev. 2001, 101 (12), 3611–3660. 10.1021/cr9901236. 11740917

[ref50] Heuts J. P. A.; Roberts G. E.; Biasutti J. D. Catalytic Chain Transfer Polymerization: An Overview. Aust. J. Chem. 2002, 55 (7), 381–398. 10.1071/CH02098.

[ref51] Heuts J. P. A.; Smeets N. M. B. Catalytic Chain Transfer and Its Derived Macromonomers. Polym. Chem. 2011, 2 (11), 2407–2423. 10.1039/c1py00224d.

[ref52] Slavin S.; McEwan K.; Haddleton D. M. Cobalt-Catalyzed Chain Transfer Polymerization: A Review. Polymer Science: a Comprehensive Reference 2012, 3, 249–275. 10.1016/B978-0-444-53349-4.00068-6.

[ref53] Huybrechts J.; Bruylants P.; Kirshenbaum K.; Vrana J.; Snuparek J. New Applications of Catalytic Chain Transfer Polymerization to Waterborne Binders for Automotive Paint Systems. Prog. Org. Coat. 2002, 45 (2), 173–183. 10.1016/S0300-9440(02)00042-5.

[ref54] Suddaby K. G.; Haddleton D. M.; Hastings J. J.; Richards S. N.; O’Donnell J. P. Catalytic Chain Transfer for Molecular Weight Control in the Emulsion Polymerization of Methyl Methacrylate and Methyl Methacrylate–Styrene. Macromolecules 1996, 29 (25), 8083–8091. 10.1021/ma960528h.

[ref55] Thomson M. E.; Smeets N. M. B.; Heuts J. P. A.; Meuldijk J.; Cunningham M. F. Catalytic Chain Transfer Mediated Emulsion Polymerization: Compartmentalization and Its Effects on the Molecular Weight Distribution. Macromolecules 2010, 43 (13), 5647–5658. 10.1021/ma100622b.

[ref56] Booth J. R.; Davies J. D.; Bon S. A. F. ω-Unsaturated Methacrylate Macromonomers as Reactive Polymeric Stabilizers in Mini-Emulsion Polymerization. Polym. Chem. 2022, 13 (10), 1335–1349. 10.1039/D1PY01664D.

[ref57] Shegiwal A.; Wemyss A. M.; Schellekens M. A. J.; de Bont J.; Town J.; Liarou E.; Patias G.; Atkins C. J.; Haddleton D. M. Exploiting Catalytic Chain Transfer Polymerization for the Synthesis of Carboxylated Latexes via Sulfur-Free RAFT. J. Polym. Sci. A Polym. Chem. 2019, 57 (3), E1–E9. 10.1002/pola.29302.

[ref58] Zhang X.; Boissé S.; Zhang W.; Beaunier P.; D’Agosto F.; Rieger J.; Charleux B. Well-Defined Amphiphilic Block Copolymers and Nano-Objects Formed in Situ via RAFT-Mediated Aqueous Emulsion Polymerization. Macromolecules 2011, 44 (11), 4149–4158. 10.1021/ma2005926.

[ref59] Ni P.; Zhu X.; Zhou X. Kinetics and Colloidal Stability of Raft/Miniemulsion Polymerization of MMA Using Comblike Polymeric Surfactants. ACS Symp. Ser. 2009, 1024, 293–302. 10.1021/bk-2009-1024.ch019.

[ref60] Krstina J.; Moad C. L.; Moad G.; Rizzardo E.; Berge C. T.; Fryd M. A New Form of Controlled Growth Free Radical Polymerization. Macromol. Symp. 1996, 111 (1), 13–23. 10.1002/masy.19961110104.

[ref61] D’Agosto F.; Rieger J.; Lansalot M. RAFT-Mediated Polymerization-Induced Self-Assembly. Angewandte Chemie - International Edition 2020, 59 (22), 8368–8392. 10.1002/anie.201911758. 31584738

[ref62] Zhang W.; D’Agosto F.; Boyron O.; Rieger J.; Charleux B. One-Pot Synthesis of Poly(Methacrylic Acid-Co-Poly(Ethylene Oxide) Methyl Ether Methacrylate)-b-Polystyrene Amphiphilic Block Copolymers and Their Self-Assemblies in Water via RAFT-Mediated Radical Emulsion Polymerization. A Kinetic Study. Macromolecules 2011, 44 (19), 7584–7593. 10.1021/ma201515n.

[ref63] Charleux B.; Delaittre G.; Rieger J.; D’Agosto F. Polymerization-Induced Self-Assembly: From Soluble Macromolecules to Block Copolymer Nano-Objects in One Step. Macromolecules 2012, 45 (17), 6753–6765. 10.1021/ma300713f.

[ref64] Lotierzo A.; Schofield R. M.; Bon S. A. F. Toward Sulfur-Free RAFT Polymerization Induced Self-Assembly. ACS Macro Lett. 2017, 6 (12), 1438–1443. 10.1021/acsmacrolett.7b00857. 35650808

[ref65] Rieger J.; Osterwinter G.; Bui C.; Stoffelbach F.; Charleux B. Surfactant-Free Controlled/Living Radical Emulsion (Co) Polymerization of n-Butyl Acrylate and Methyl Methacrylate via RAFT Using Amphiphilic Poly (Ethylene Oxide)-Based Trithiocarbonate Chain Transfer Agents. Macromolecules 2009, 42 (15), 5518–5525. 10.1021/ma9008803.

[ref66] Schreur-Piet I.; Heuts J. P. A. Amphiphilic Statistical Copolymers from Catalytic Chain Transfer as Reactive Surfactants in Emulsion Polymerization. ACS Appl. Polym. Mater. 2021, 3 (9), 4616–4624. 10.1021/acsapm.1c00715.

[ref67] Schreur-Piet I.; van Herk A. M.; Laven J.; Heuts J. P. A. Synthesis and Rheological Characterization of Latexes Stabilized by Methacrylic Acid Containing Macromonomers. Ind. Eng. Chem. Res. 2019, 58 (46), 21105–21117. 10.1021/acs.iecr.9b02794.

[ref68] Schreur-Piet I.; Heuts J. P. A. In Situ Stabilizer Formation from Methacrylic Acid Macromonomers in Emulsion Polymerization. Polym. Chem. 2017, 8 (43), 6654–6664. 10.1039/C7PY01583F.

[ref69] Patton T. C. Paint Flow and Pigment Dispersion: A Rheological Approach to Coating and Ink Technology, 2nd ed.; John Wiley & Sons: New York, 1979.

[ref70] Abdollahi M. Effect of Carboxylic Acid Monomer Type on Particle Nucleation and Growth in Emulsifier-Free Emulsion Copolymerization of Styrene-Carboxylic Acid Monomer. Polym. J. 2007, 39 (8), 802–812. 10.1295/polymj.PJ2006246.

[ref71] Mewis J.; Wagner N. J. Colloidal Suspension Rheology, 1st ed.; Cambridge University Press: Cambridge, 2012.

[ref72] Goodwin J. W.; Hughes R. W. Rheology for Chemists, 2nd ed.; Royal Society of Chemistry: Cambridge, 2008.

[ref73] English R. J.; Laurer J. H.; Spontak R. J.; Khan S. A. Hydrophobically Modified Associative Polymer Solutions: Rheology and Microstructure in the Presence of Nonionic Surfactants. Ind. Eng. Chem. Res. 2002, 41, 6425–6435. 10.1021/ie020409s.

[ref74] Andrews R. J.; Grulke E. A. Polymer Handbook. In Polymer Handbook; Brandrup J., Immergut E. H., Grulke E. A., Eds.; John Wiley & Sons: New York, 2003; p VI/194–277.

[ref75] Tóth J. Thermodynamical Correctness of Gas/Solid Adsorption Isotherm Equations. J. Colloid Interface Sci. 1994, 163 (2), 299–302. 10.1006/jcis.1994.1107.

[ref76] Donohue M.; Aranovich G. L. Classification of Gibbs Adsorption Isotherms. Adv. Colloid Interface Sci. 1998, 76, 137–152. 10.1016/S0001-8686(98)00044-X.

[ref77] Liu Y.; Soer W.-J.; Scheerder J.; Satgurunathan G.; Keddie J. L. Water Vapor Sorption and Diffusion in Secondary Dispersion Barrier Coatings: A Critical Comparison with Emulsion Polymers. ACS Appl. Mater. Interfaces 2015, 7 (22), 12147–12157. 10.1021/acsami.5b02446. 25985183

[ref78] Voogt B.; Huinink H.; van de Kamp-Peeters L.; Erich B.; Scheerder J.; Venema P.; Adan O. Hydroplasticization of Latex Films with Varying Methacrylic Acid Content. Polymer 2019, 166, 206–214. 10.1016/j.polymer.2019.01.074.

[ref79] Standard Test Methods for Hardness of Organic Coatings by Pendulum Damping Tests ASTM D4366, ASTM International; 2016.

[ref80] Standard Test Methods for Measuring Adhesion by Tape Test ASTM D3359, ASTM International.

[ref81] Berry J. D.; Neeson M. J.; Dagastine R. R.; Chan D. Y. C.; Tabor R. F. Measurement of Surface and Interfacial Tension Using Pendant Drop Tensiometry. J. Colloid Interface Sci. 2015, 454, 226–237. 10.1016/j.jcis.2015.05.012. 26037272

[ref82] Crank J. J.; Park G. S.(Ed.). Diffusion in Polymers, 2nd ed.; Academic Press: London, 1975.

[ref83] Cannon L. A.; Pethrick R. A. Effect of the Glass-Transition Temperature on Film Formation in 2-Ethylhexyl Acrylate/Methyl Methacrylate Emulsion Copolymers. Macromolecules 1999, 32 (22), 7617–7629. 10.1021/ma990273i.

[ref84] Marrion A. R.; Cameron C.; Port A. B. Film Formation. In The Chemistry and Physics of Coatings; Marrion A. R., Ed.; Royal Society of Chemistry: Cambridge, 2004; pp 46–63.

[ref85] Butt H.-J.; Gerharz B. Imaging Homogeneous and Composite Latex Particles with an Atomic Force Microscope. Langmuir 1995, 11, 4735–4741. 10.1021/la00012a026.

[ref86] Lu L.; Duan H.; Li J.; Qi D. Film-Formation and Binder-Free Pigment Printing of Fluorosilicone-Modified Polyacrylate/Pigment Hybrid Latex: Effect of Cross-Linking Degree. ACS Appl. Polym. Mater. 2023, 5 (3), 1871–1881. 10.1021/acsapm.2c01960.

[ref87] Palmer T. R.; van der Kooij H. M.; Abu Bakar R.; Duewel M.; Greiner K.; McAleese C. D.; Couture P.; Sharpe M. K.; Smith R. W.; Keddie J. L. How Particle Deformability Influences the Surfactant Distribution in Colloidal Polymer Films. Langmuir 2022, 38 (41), 12689–12701. 10.1021/acs.langmuir.2c02170. 36194469 PMC9583616

[ref88] Henke M.; Lis B.; Krystofiak T. Evaluation of Surface Roughness Parameters of HDF for Finishing under Industrial Conditions. Materials 2022, 15 (18), 6359–6379. 10.3390/ma15186359. 36143669 PMC9506473

[ref89] Feng J.; Berger K. R.; Douglas E. P. Water Vapor Transport in Liquid Crystalline and Non-Liquid Crystalline Epoxies. J. Mater. Sci. 2004, 39, 3413–3423. 10.1023/B:JMSC.0000026944.85440.f3.

[ref90] Jalilian E.; Terryn H.; Van Assche G. Water Permeation in Coatings. J. Coat. Technol. Res. 2020, 17 (6), 1437–1445. 10.1007/s11998-020-00377-6.

[ref91] Sangaj N. S.; Malshe V. C. Permeability of Polymers. Prog. Org. Coat. 2004, 50 (1), 28–39. 10.1016/j.porgcoat.2003.09.015.

[ref92] Soleimani M.; Haley J. C.; Lau W.; Winnik M. A. Effect of Hydroplasticization on Polymer Diffusion in Poly(Butyl Acrylate-Co-Methyl Methacrylate) and Poly(2-Ethylhexyl Acrylate-Co-Tert-Butyl Methacrylate) Latex Films. Macromolecules 2010, 43 (2), 975–985. 10.1021/ma9020483.

[ref93] Baukh V.; Huinink H. P.; Adan O. C. G.; Erich S. J. F.; Van Der Ven L. G. J. Predicting Water Transport in Multilayer Coatings. Polymer 2012, 53 (15), 3304–3312. 10.1016/j.polymer.2012.05.043.

[ref94] Rueckel M.; Gerst M.; Wolf T.; Willerich I. Fluorescence Imaging for Studying the Water Uptake and Drying Process of Polymer Films. Prog. Org. Coat. 2023, 179, 107548–110759. 10.1016/j.porgcoat.2023.107548.

