# Supplementary material for: The Effect of Macromonomer Surfactant Microstructure on Aqueous Polymer Dispersion and Derived Polymer Film Properties
Source: Biomacromolecules. 2024 Jun 11;25(7):4203–14. doi: 10.1021/acs.biomac.4c00292 (PMC11238338; doi:10.1021/acs.biomac.4c00292)
Supplement: Supplementary file 1 — bm4c00292_si_001.pdf [file bm4c00292_si_001.pdf]

Supporting Information for

The effect of macromonomer surfactant microstructure on aqueous polymer dispersion and derived polymer film properties

Ingeborg Schreur-Piet and Johan P.A. Heuts

Department of Chemical Engineering & Chemistry and Institute for Complex Molecular Systems, Eindhoven University of Technology, PO Box 513, 5600 MB Eindhoven, The Netherlands

Contents

1. Introduction 2
2. Characterization techniques 2
   Characterization of latexes 2
   Characterization of emulsion polymers 3
   Characterization of polymer coatings 5
3. Overview results per latex 7
4. Comparison of DLS data 18
5. AFM images and surface roughness 19
   Overview of AFM images 19
   Comparison of surface roughness 21
6. Overview of SEM images 22
7. Comparison of SEM and Cryo-TEM images 23
8. Rheology data 25
9. Overview of static and dynamic contact angle measurements 26
10. Comparison of DVS sorption plots 27
11. Additional results of emulsion polymerization 28

1. Introduction

In the supplementary information we will first describe the characterization techniques used to analyze the latexes, the emulsion polymers, and the coatings. Next, we will show the analysis results per latex (Figure S2 to S11). Furthermore, we will give an overview of the DLS data for all latexes analyzed using different models (Table S2), the roughness and goniometry results using the four different preparation methods for all coatings (Tables S3 and S4, respectively). Next, we will give an overview of images obtained using AFM (Figure S12 and S13) and SEM (Figure S14), and subsequently we will show some remarkable images of cryo-TEM and SEM for some of the latexes (Figure S15 and S16). Finally, we will show additional rheology results of the latexes (Figure S17 and S18) and DVS results (Figure S19 and S20).

2. Characterization techniques

Characterization of latexes

Dynamic Light Scattering (DLS)

The particle size distributions of the latexes were measured by Dynamic Light Scattering (DLS) on an AntonPaar Litesizer^{TM} 500 at 25°C. The used laser is a 40 mW laser with a wavelength of 628 nm. The angle of incident-to-scattered light is 175° (backscatter). The dispersions were diluted with a sodium carbonate buffer solution ($pH = 10$) to ca. 0.01 wt% and measured three times at a scattering angle of 175° (backscattering) using an omega cuvette suitable for particle size and zeta potential measurements. Both the CUMULANT (called 'general advanced' in the used AntonPaar software) and CONTIN algorithms were used to analyze the second order autocorrelation functions. CONTIN results are only shown when different from the CUMULANT results. The mean diameter was evaluated from the Stokes-Einstein equation for spheres. D_n is number-average diameter, D_v is volume average mean diameter, D_I is intensity average mean diameter and D_H is hydrodynamic diameter. Measurement range of the instrument is 0.1 to 10000 nm. To determine the zeta potential of the latexes an electric field of 200 V was applied on the same cuvette. The velocity of the particles was determined through Phase Analysis Light Scattering (PALS) and the Smoluchowski approximation with a Henry factor of 1.5 was used.

Titration procedure: to measure the influence of the *pH*, the latex was diluted 1000x with demineralized water, the start *pH* was adjusted above 10.0 with 1 M NaOH and subsequently the latex was titrated over a *pH* range 10.0-2.0 using a 0.10 M solution of HCl. Zeta potentials and hydrodynamic diameters were measured using the above-mentioned instrument settings.

Rheology

Rheological measurements on the latexes were performed at 20 °C using an AntonPaar Physica MCR 301 Rheometer using the concentric cylinder CC27 system (inner and outer diameters are 26.66 mm and 28.92 mm, respectively) in the auto optimization mode. To get a more quantitative insight into the rheological properties of the prepared latexes we measured the viscosities as a function of the shear rate between 0.001 and 100 s^{-1}; for each measurement point the shear rate was kept constant until the viscosity reached a constant value. We also probed the mechanical microstructure by measuring the dynamic moduli *G*' and *G*'' for a period of 1000 s at a frequency of 6.3 rad·s^{-1} and stress of 0.001 Pa (*i.e.*, a dynamic time sweep). To check the recoverability of the structure after such time sweep, the sample was agitated (using a shear rate of 100 s^{-1} for 100 s), after which another dynamic time sweep was recorded. The linear viscoelastic region was determined by performing a strain sweep experiment at angular frequency of 6.3 rad·s^{-1}. A stress of 0.001 Pa was chosen because this stress was sufficiently high to effectively suppress noise and small enough to stay in the linear region.

Characterization of emulsion polymers

Differential Scanning Calorimetry (DSC)

Glass transition temperatures (T_g) of the macromonomeric surfactants and the dried emulsion polymers were determined on a Waters-TA Instruments DSC-Q2000. Dry samples (5-10 mg) were heated from room temperature using a heat rate of 10 °C min^{-1} under dry nitrogen (50 cm^3 min^{-1}) to 200 °C; subsequently cooled to -90 °C using a cooling rate of 5 °C min^{-1} and heated again to 200 °C at a rate of 10 °C min^{-1} (standard heat-cool-heat cycle). The T_g was determined using TA Instruments Trios software, taking the midpoint of the transition in the second heating run as the value for T_g. The glass transition temperatures of the copolymers were also estimated using the Fox equation[1] in combination with the homopolymer T_g data listed in Table S1.

$$\frac{1}{T_g} = \sum \frac{w_i}{T_g}. \qquad \textbf{(Eq. S1)}$$

Table S1 Literature values for the glass transition temperature of the homopolymers[1]

	T_g (°C)
Poly (butyl acrylate)	-49
Poly (methacrylic acid)	228
Poly (methyl methacrylate)	105
Poly (butyl methacrylate)	20
Poly (lauryl methacrylate)	-65

Dynamic vapor sorption (DVS)

Moisture sorption isotherms of the bulk polymers were obtained using a dynamic vapor sorption analyzer (DVS Adventure, Surface Measurements Systems). It uses an ultrasensitive microbalance to measure the mass of the sample as a function of time and relative humidity (*RH*) at a fixed temperature. The inlet pressure had a constant value of 2.0 bar. The resolution of the balance is 0.1μg using a weight between 5 and 100 mg. Controlled humidity was maintained using laminar flow with wet−dry vapor mixing at a constant mass flow rate (200 mL/min) avoiding mass transfer limitations. In an adsorption experiment, the sample was initially held at a RH of 0% at a temperature of 20°C until the mass was stable over time (*dm/dt* <0.001%), indicating equilibrium. This mass was used as M_0. The *RH* was increased to 95% in steps of 10%. At each RH, the mass was recorded as a function of time until it reached equilibrium (M_∞ at *dm/dt* <0.001%). In "characterization of polymer coatings" we will discuss the characterization using DVS of free-standing polymer films.

Electron Microscopy

SEM analyses were performed on a FEI Quanta 3D FEG (ETD; 5kV; 27 pA). All latex samples were diluted ca. 1000x and coated with a 10 nm gold layer with a Quorum Q150T sputter coater prior to imaging. The average diameter of the latex particles (count > 100) was measured manually from the SEM images.

Vitrified thin films for Cryo-TEM analysis were prepared using an automated vitrification robot (FEI, Vitrobot Mark IV) by plunge vitrification in liquid ethane. Before vitrification, a 200-mesh copper grid covered with a Quantifoil R 2/2 holey carbon film (Quantifoil Micro Tools GmbH) was surface plasma treated for 40 seconds using a Cressington 208 carbon coater. Cryo-TEM imaging was carried out on the TITAN (Thermo Fisher, previously FEI), equipped with a field emission gun (FEG), a post-column Gatan imaging filter (model 2002) and a post-GIF 2k × 2k Gatan CCD camera (model 794). The microscope was operated at 300 kV acceleration voltage in bright-field TEM mode with zero-loss energy filtering.

Size Exclusion Chromatography (SEC)

SEC was carried out on a Waters Alliance system equipped with a Waters 2695 separation module, a Waters 2414 refractive index detector (40 °C), a Waters 2487 dual UV absorbance detector, a PSS SDV 5 μm bead size guard column followed by two PSS SDV 5 μm bead size linearXL columns in series (300 x 8 mm) at 40 °C. Tetrahydrofuran (THF stabilized with BHT, Biosolve) with 1 v/v-% acetic acid and 1 wt% toluene was used as eluent at a flow rate of 1.0 mL min^{-1}. The system was calibrated using polystyrene standards (Polymer Laboratories, M_n = 580 - 7.1·10^6 g mol^{-1}).

Characterization of polymer coatings

Atomic Force Microscopy (AFM)

AFM (topography and phase contrast) was carried out on thin coatings (60 μm) with a NT-MDT Solver operated in non-contact tapping mode (used tip: HA_NC[2], K=130 kHz, F=4.4 Nm^{-1}) under ambient conditions. The ten-point height (R_z) and roughness (R_a) of the surface at evaluation lengths (L) of 20 μm, 5 μm and/or 1 μm were calculated using Nova software according to ISO standard 21920.[2] R_z is the average value of the heights of the five highest profile heights and the depths of the five deepest valleys within the evaluation length. R_a is the arithmetic average of the absolute values of the profile heights over the evaluation length. Z(x) is the profile height function. $R_a = \left(\frac{1}{L}\right)\int_0^L |Z(x)|dx$. **(Eq. S2)**

Cross hatch Adhesion test

Hardness was measured for thin coatings (60 μm) on an aluminum substrate, coatings were dried at 20 °C (Prep A). Two series of 5 parallel cuts, 2 mm apart of approximately 20 mm long were made with an Elcometer 1542 Cross Hatch Adhesion tester, through the coating to the substrate. The second cut was made perpendicular to the first cut to create a lattice pattern on the coating. The sample was brushed to remove debris. Adhesive tape was placed on the lattice, rubbed firmly, and pulled back at an angle of 180°. The surface was examined with an optical stereomicroscope. Test was performed according to ASTM D3359-23B.[3]

Dynamic mechanical analysis (DMA)

DMA measurements of cast films dried at 20 °C in aluminum cup with a minimum film thickness of 500 μm, were performed using an AntonPaar Physica MCR 301 Rheometer with the parallel plate with a diameter of 8 mm. Storage and loss moduli and loss angle were measured during a temperature sweep from 5 °C to 180 °C, with a constant force of 5 N and an increasing strain over the temperature trajectory from 0.1% to 1%.

Goniometry: contact angle and surface tension measurements

The contact angles (CA) of the thin coatings (60 μm) were measured by an OCA15Pro goniometer from DataPhysics using a DataPhysics SCA software-controlled system. The static contact angles of the films were measured using the sessile drop-method.[4] Before the measurements a Hamilton 500 μL syringe was filled with demineralized water. A water droplet of 2 μL was suspended at the end of a syringe needle. The syringe was moved down slowly until the pendant drop touched the surface, transferring the droplet to the sample film. Considering the possible variations in treatment and surface roughness of the sample films, measurements were carried out on ten spots per sample. With the automatic evaluation function of the software, the mean static water contact angles were obtained using an elliptical fit to the

drop shape. The advancing and receding contact angle (ACA and RCA, respectively) were measured with the needle in sessile drop-method.[4] The syringe was placed 1 mm above the surface, 1 μL water was added with the syringe, the position of the needle was corrected to the middle of the drop, then the water was dosed at a dosing rate of 0.05 μL/s until a droplet volume of 18 μL, after a delay period of 20 s the water was sucked with the needle at the same rate. The contact angles were measured continuously. The diameter of the syringe needle was 0.52 mm.

To determine whether free surface-active groups were present on the surfaces of the coatings, surface tension measurements were performed on the same instrument. 20 μL of water was placed on the surface of a film using the above-mentioned needle in sessile drop-method. The water droplet was sucked and replaced on the surface twice before the droplet was sucked with an empty syringe with a larger needle diameter (d = 1.65 mm). The coating was moved down, and the droplet was dosed slowly from the syringe in the air, and just before the droplet reached its maximum size and drops, the surface tension was determined using the automatic shape recognition function of the software, using the Young-Laplace equation.[5]

Pendulum hardness (König)

König Hardness was measured for thin coatings (60 μm) on a glass substrate, coatings were dried at 20 °C (Prep A). A König pendulum hardness tester (Erichsen, Model299) was used to figure out the surface hardness. The pendulum hardness of the surfaces was measured using respect to the pendulum oscillation time from 6° to 3° at 23 ± 1 °C and 50 ± 2 %*RH*. The pendulum hardness test is based on the principle that the harder a measured surface, the greater the amplitude time of pendulum oscillation. The König pendulum consists of a triangular open framework with an adjustable counterpoise weight of 200 ± 0.2 g. The pendulum pivots on two bearings of 5 mm diameter that rest on the test surface. The instrument was calibrated using a glass substrate, giving 100 swings in 140 ± 2 s. Test was performed according to ASTM D4366.[6]

Gloss

Gloss measurements (gloss, haze, and reflection) were performed on thin coatings (60 μm) on an aluminum substrate using an Elcometer 480 Triple angle glossmeter. The coatings were dried at 20 °C (Prep A). The mean value of 5 measurements was calculated at different angles. In this work the results for gloss at a reflection angle of 85° are reported. To check whether multiple reflections had increased the gloss values also the percentage reflection of the incident light was measured. Test according to ASTM D523-14.[7]

Diffusion coefficient and permeability with DVS

The diffusion through and permeability of cast films (thickness between 220 and 760 µm, and vacuum oven dried at 60 °C) were measured using a Payne-type diffusion cell (weighing cup method). The design of this cell is shown in Figure S1.

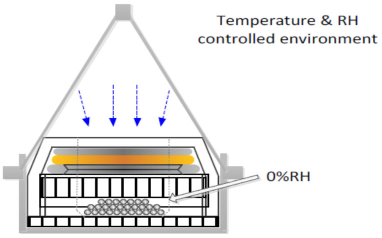

Figure S1 Experimental set-up for a moisture vapour transmission rate measurement using the dry cup method (Payne cell) at T=25 °C; inside cell RH = 0%, outside cell RH = 95%.

The cell has an opening diameter of 4.4 mm on the top, providing an area of 15.54 mm^2 for moisture transport. The thickness of the film (d) was measured with a micrometer. The sorption temperature was 25 °C. The films were pre-dried for 4 hours and subsequently measured at 0%, 95%, and 0% RH. At each RH, the mass (M) was recorded as a function of time until it reached equilibrium (M_∞ at dm/dt <0.0001%). Moisture vapor transmission rate ($MVTR$) describes the rate of water vapor permeating through a test specimen into the headspace volume of the cell which differs in relative humidity. For the calculations an RH of 95% outside the cell and 0% inside cell was used. The permeability (P) was calculated from the measured $MVTR$ and the film thickness (d): $P = MVTR \cdot d$ **(Eq. S3)**

The diffusion coefficient (D_{dif}) was calculated using the software from the initial slope of a plot of M_t/M_∞ against $t^{0.5}/d$ using the following equation for Fickian diffusion:[8]

$$\frac{M_t}{M_\infty} = \frac{4}{d}\sqrt{\frac{D_{dif}.t}{\pi}} \qquad \textbf{(Eq. S4)}$$

3. Overview results per latex

In this section an overview of the analysis results per latex are summarized. In the subtitle the type of latex I, II or III and if applicable any sedimentation is mentioned, followed by the rheological behavior: Newtonian or shear thinning and average roughness, R_a. In the figure the particle size distribution (PSD) determined by the CUMULANT model (CONTIN model when indicated) and the Zeta potential (ζ) and the hydrodynamic diameter (D_H) as a function of the pH are shown. Furthermore, typical SEM and cryo-TEM images are shown and additionally, plots of the roughness profile with maximum and minimum values indicated and of the DSC, DMA, and SEC measurements, if available, are shown.

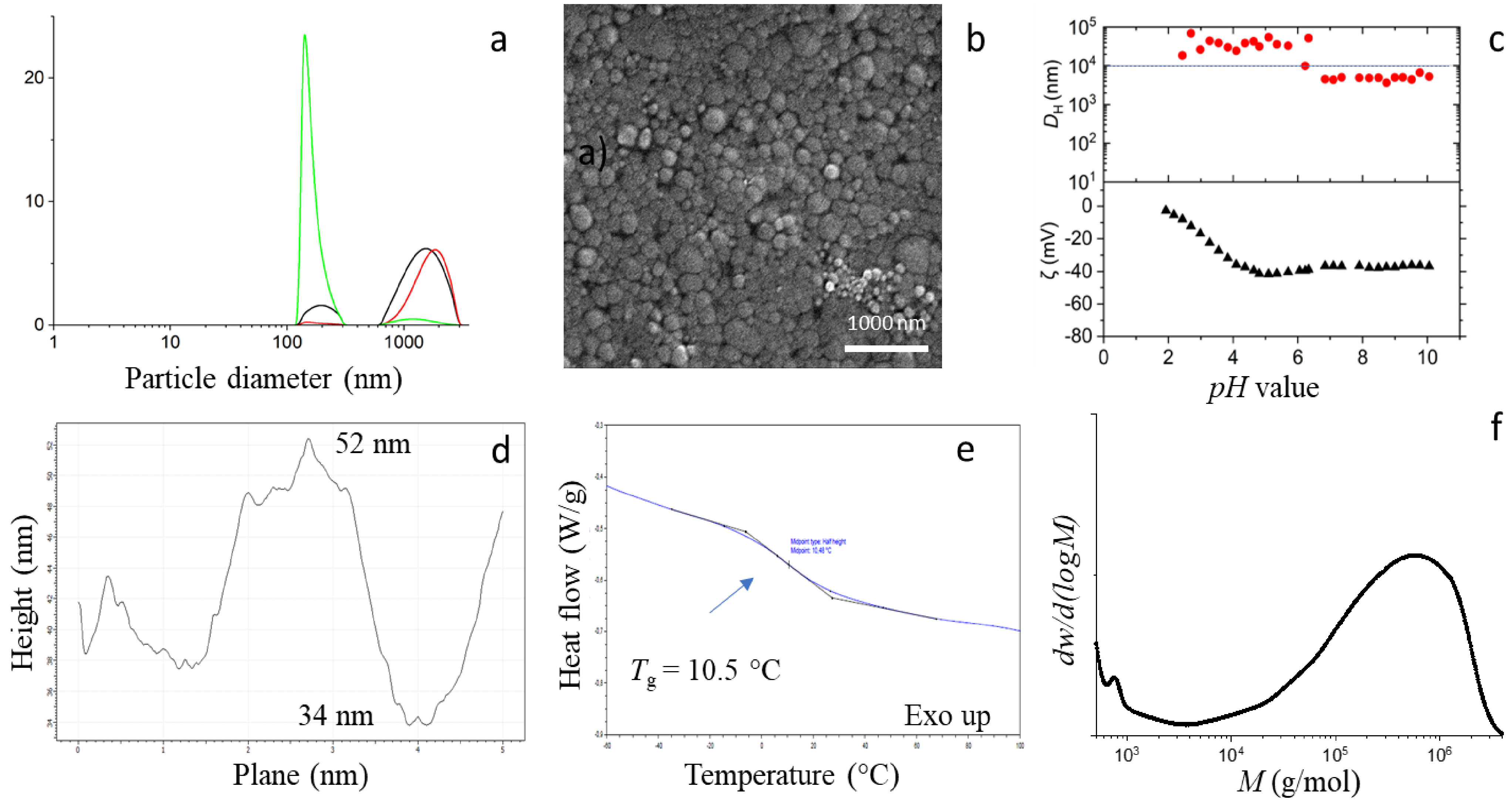

Figure S2 Overview results sL_I: latex type III, ST; a) PSD CUMULANT model, D_I (-); D_v (-) and D_n (-); b) SEM image diluted latex, bar 1000 nm; c) Zeta potential (▲) and D_H (●) as a function of the *pH*; d) AFM film roughness line profile, R_a= 5 nm; e) DSC; f) Molar mass distribution determined with SEC.

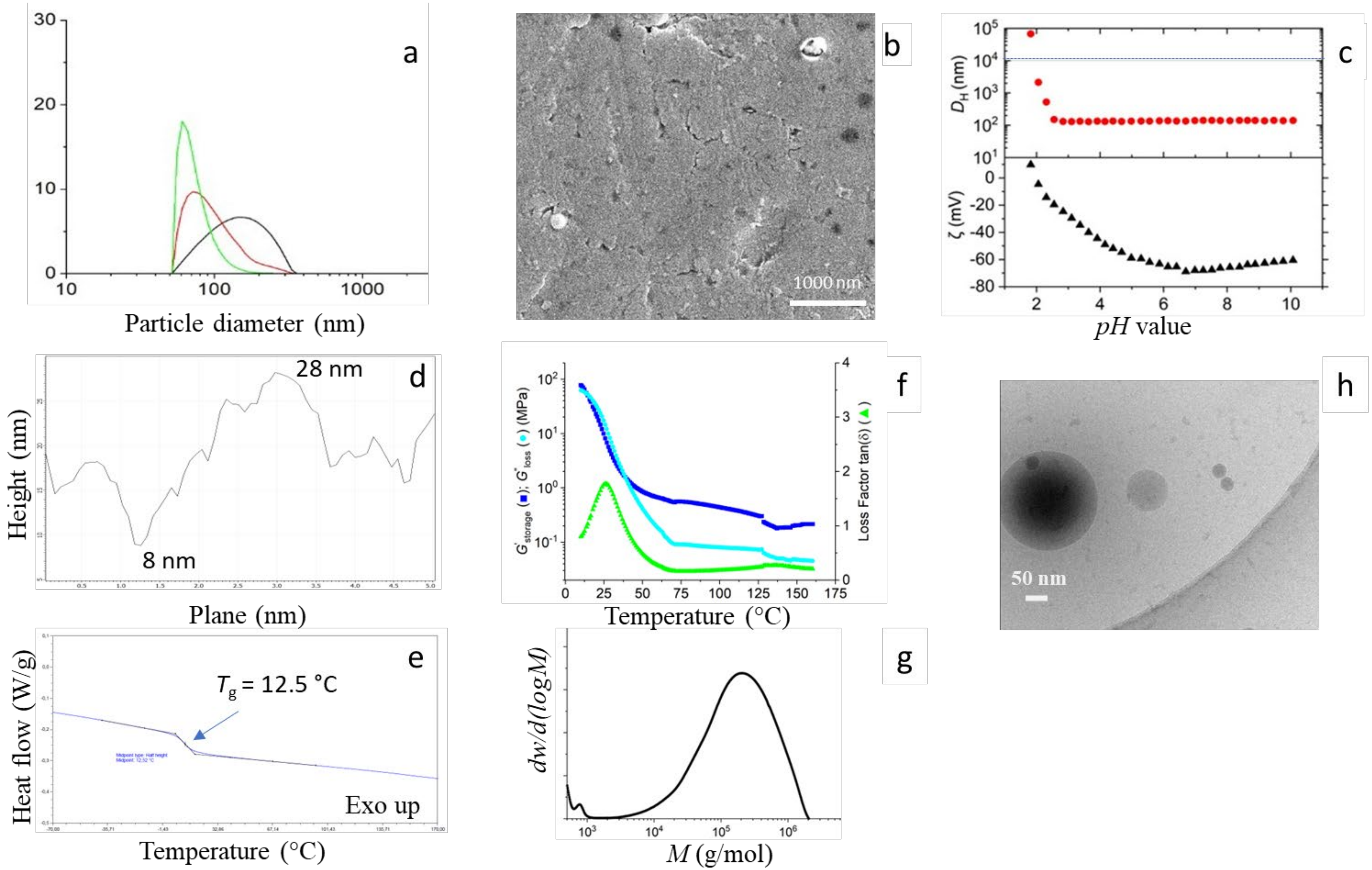

Figure S3 Overview results sL_s: latex type I, shear thinning, a) PSD CUMULANT model, D_I (-); D_v (-) and D_n (-); b) SEM image diluted latex, bar 1000 nm; c) Zeta potential (▲) and D_H (●) as a function of the *pH*; d) AFM film roughness line profile over 5 µm, R_a= 3 nm; e) DSC; f) Storage and loss moduli and loss factor in temperature sweep for cast coatings, dried at 20 °C; g) Molar mass distribution determined with SEC h) Cryo-TEM image, 70 nm particle and large "core-shell-like" particle, bar 50 nm.

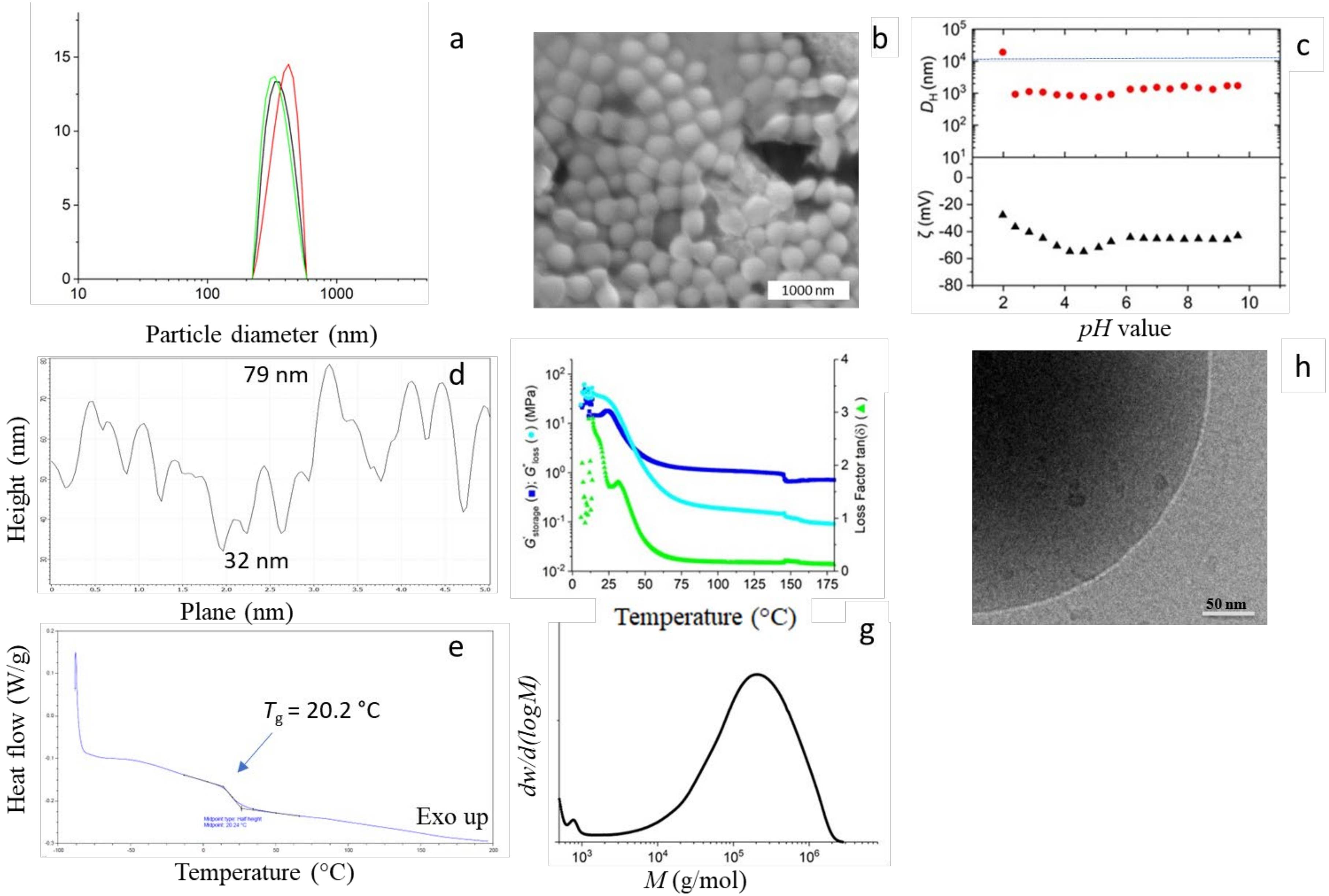

Figure S4 Overview results sB$_I$: latex type II, ST; a) PSD CUMULANT model, D_I (-); D_v (-) and D_n (-); b) SEM image diluted latex, bar 1000 nm; c) Zeta potential (▲) and D_H (●) as a function of the pH; d) AFM film roughness line profile over 5 µm, R_a= 8 nm; e) DSC; f) Storage and loss moduli and loss factor in temperature sweep for cast coatings, dried at 20 °C; g) Molar mass distribution determined with SEC h) Cryo-TEM image, bar 50 nm.

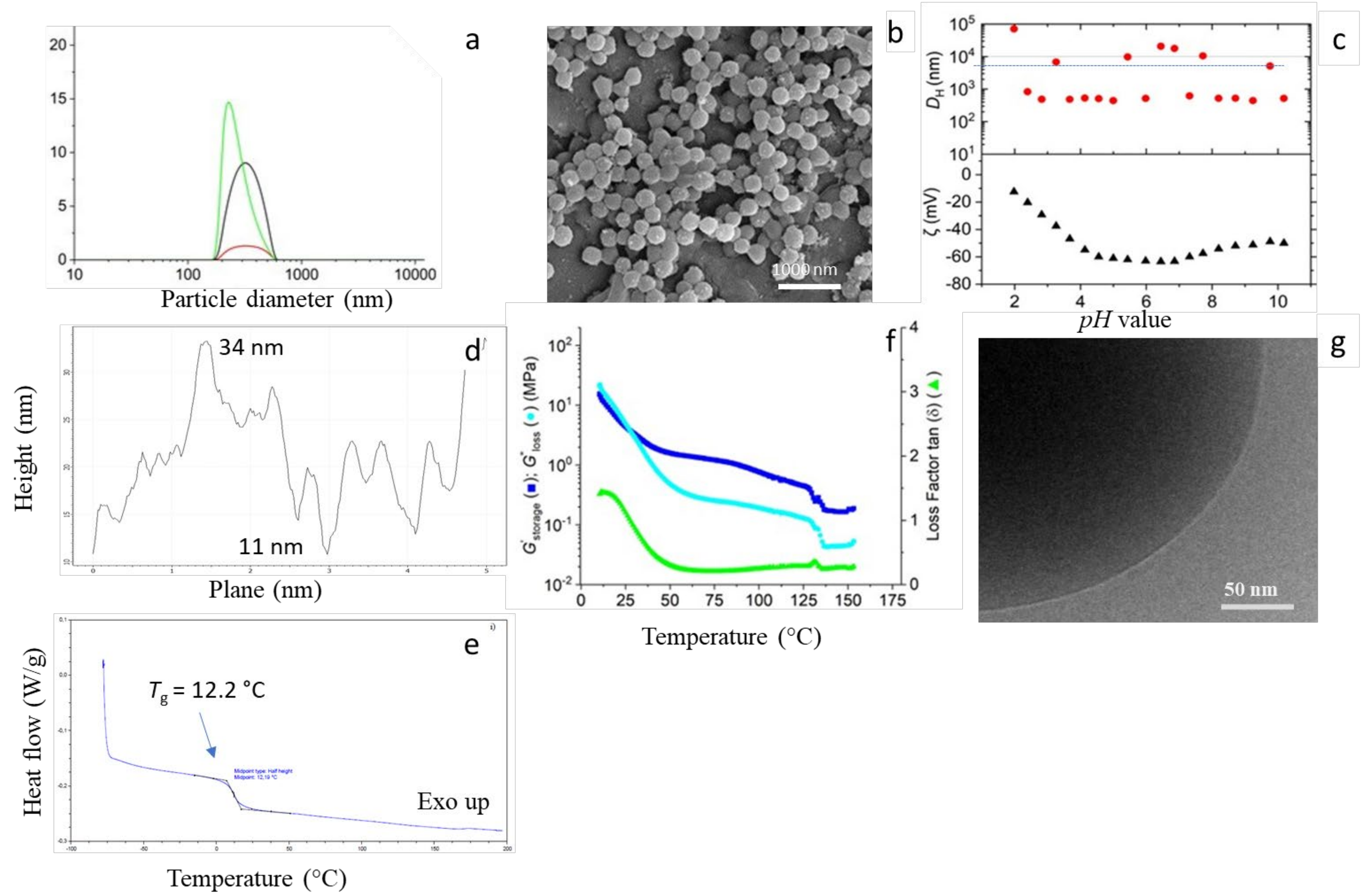

Figure S5 Overview results sB_s: latex type I, Newtonian; a) PSD CONTIN model D_I (-); D_v (-) and D_n (-); b) SEM image diluted latex, bar 1000 nm; c) Zeta potential (▲) and D_H (●) as a function of the *pH*; d) AFM film roughness line profile over 5 µm, R_a= 3 nm; e) DSC; f) Storage and loss moduli and loss factor in temperature sweep for cast coatings, dried at 20 °C; g) Cryo-TEM image, bar 50 nm.

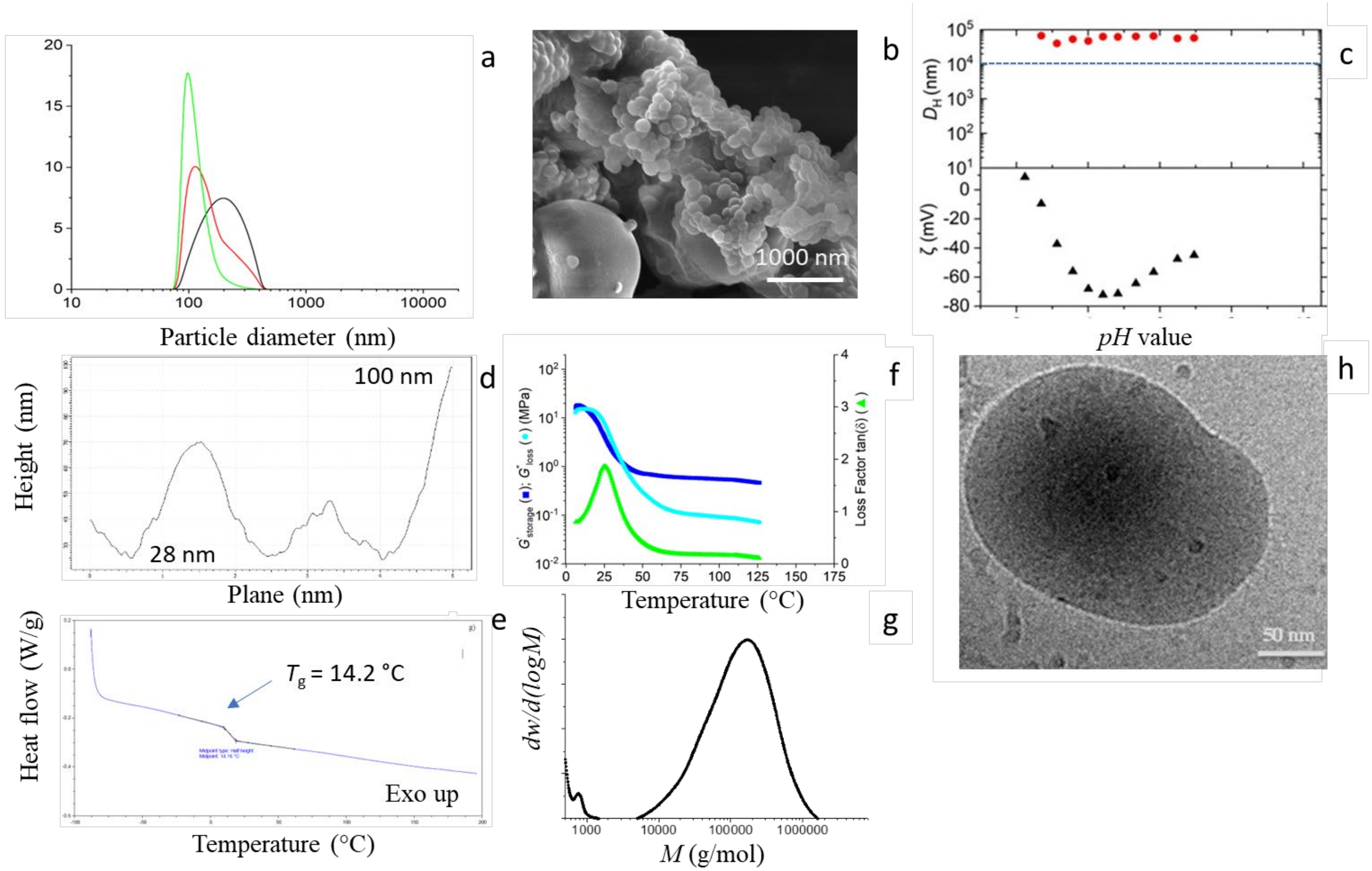

Figure S6 Overview results sM_I: latex type I, Newtonian; a) PSD CUMULANT model D_I (-); D_v (-) and D_n (-); b) SEM image diluted latex, bar 1000 nm; c) Zeta potential (▲) and D_H (●) as a function of the *pH*; d) AFM film roughness line profile over 5 µm, R_a= 7 nm; e) DSC; f) Storage and loss moduli and loss factor in temperature sweep for cast coatings, dried at 20 °C; g) Molar mass distribution determined with SEC h) Cryo-TEM image, hairy structure, bar 50 nm.

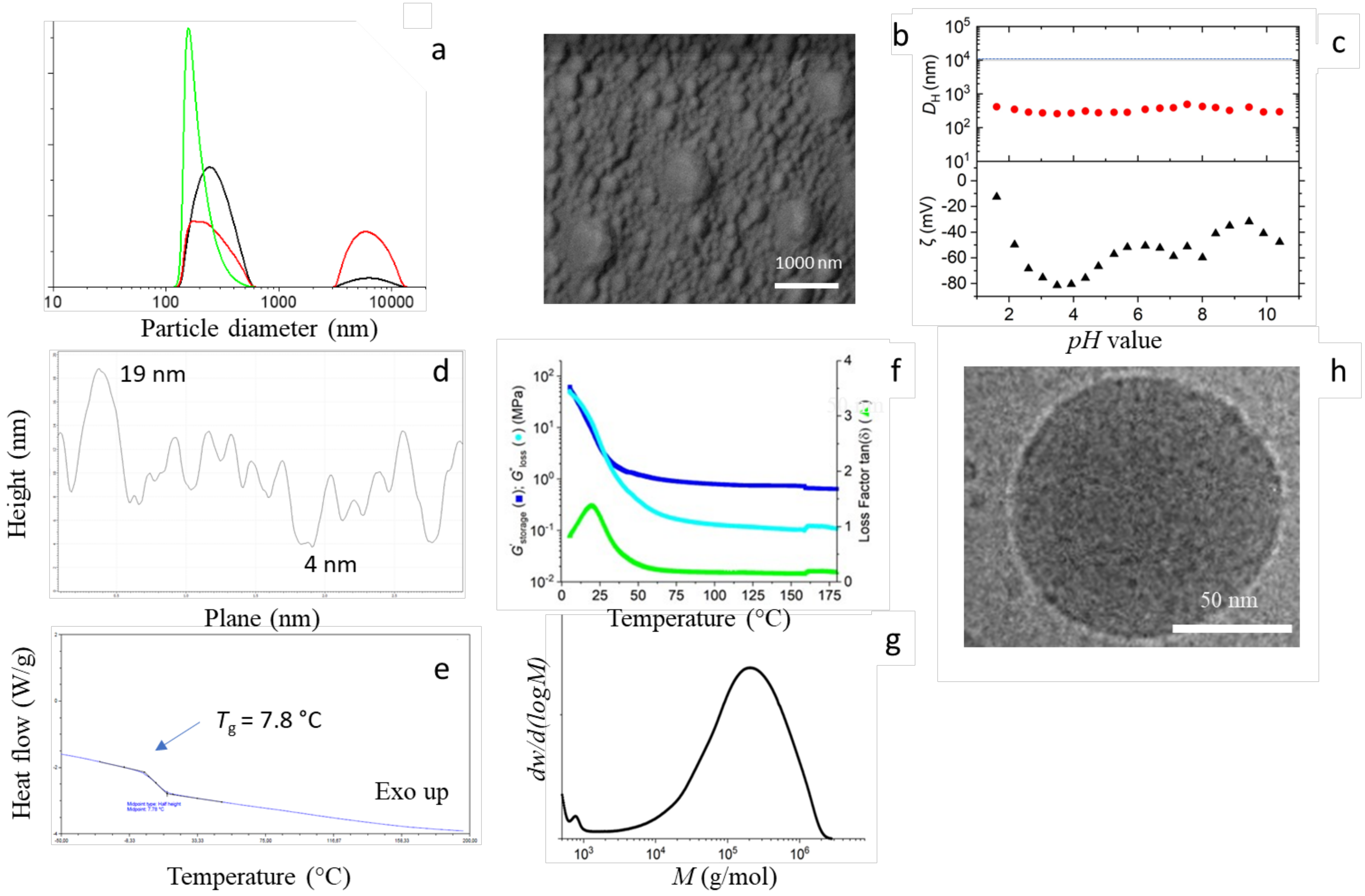

Figure S7 Overview results sM_s: latex type I, Newtonian; a) PSD CUMULANT model D_I (-); D_v (-) and D_n (-); b) SEM image diluted latex, bar 1000 nm; c) Zeta potential (▲) and D_H (●) as a function of the pH; d) AFM film roughness line profile over 5 µm, R_a= 16 nm; e) DSC; f) Storage and loss moduli and loss factor in temperature sweep for cast coatings, dried at 20 °C; g) Molar mass distribution determined with SEC; h) Cryo-TEM image, bar 50 nm.

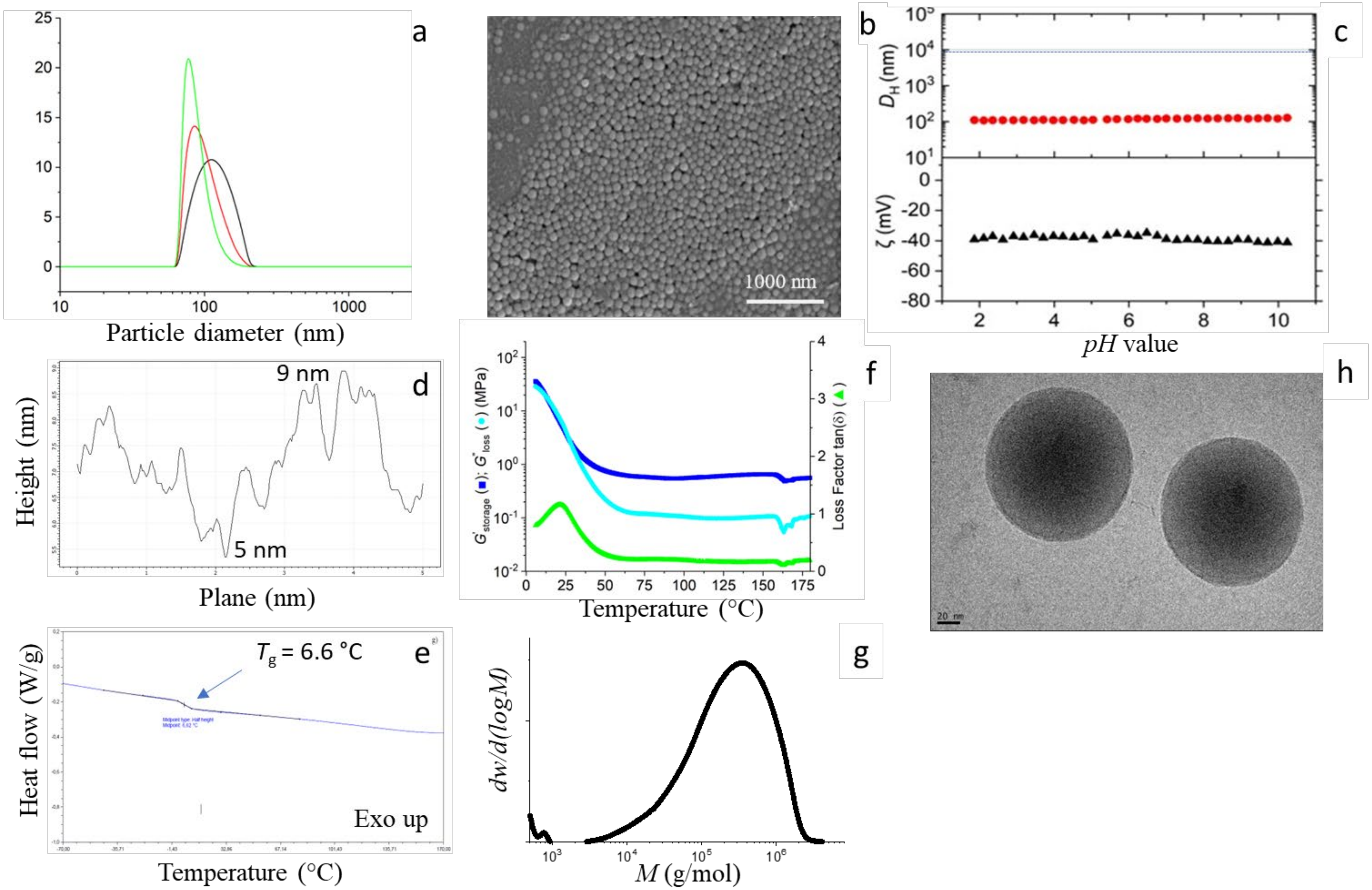

Figure S8 Overview results *b*M: latex type I, shear thinning; a) PSD CUMULANT model D_I (**−**); D_v (**−**) and D_n (**−**); b) SEM image diluted latex, bar 1000 nm; c) Zeta potential (▲) and D_H (●) as a function of the *pH*; d) AFM film roughness line profile over 5 µm, R_a= 1 nm; e) DSC; f) Storage and loss moduli and loss factor in temperature sweep for cast coatings, dried at 20 °C; g) Molar mass distribution determined with SEC h) Cryo-TEM image, bar 20 nm.

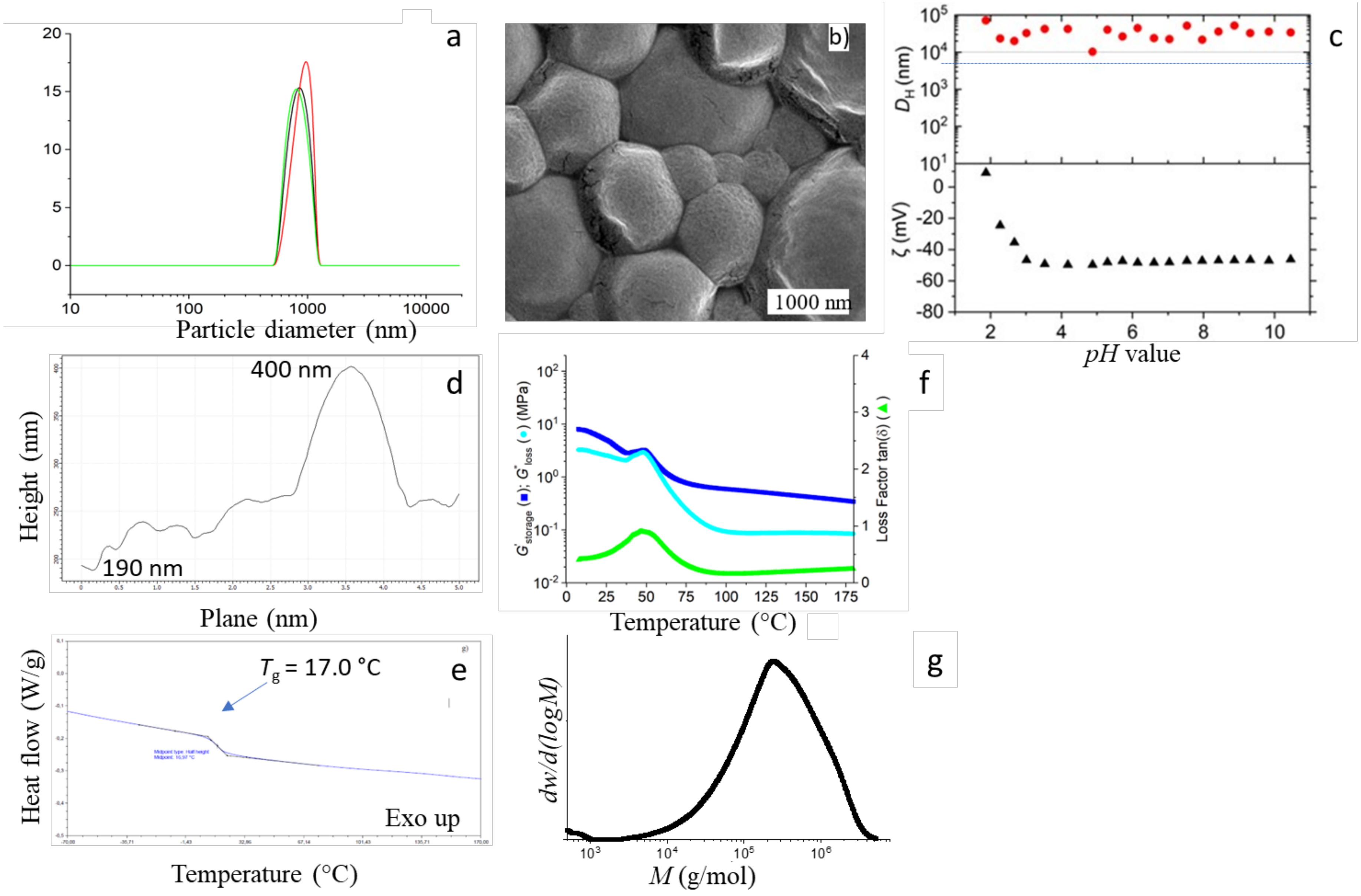

Figure S9 Overview results hA_I: latex type II, sedimentation, shear thinning; a) PSD CUMULANT model D_I (−); D_v (−) and D_n (−); b) SEM image diluted latex bar 1 μm; c) Zeta potential (▲) and D_H (●) as a function of the *pH*; d) AFM film roughness line profile over 5 μm, R_a= 63 nm; e) DSC; f) Storage and loss moduli and loss factor in temperature sweep for cast coatings, dried at 20 °C; g) Molar mass distribution determined with SEC.

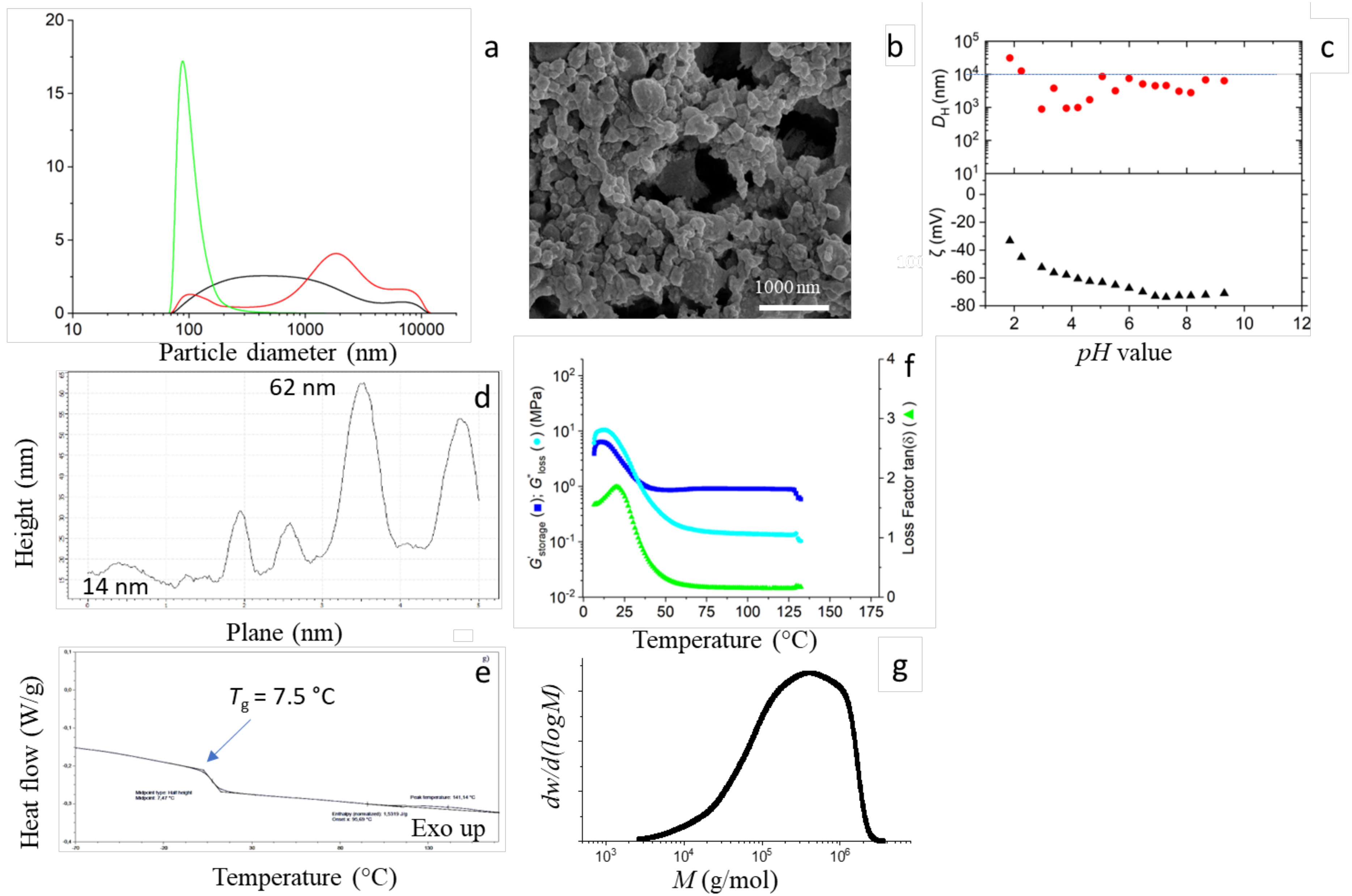

Figure S10 Overview results hA_s: latex type II, some coagulation in time, shear thinning; a) PSD CUMULANT model D_I (-); D_v (-) and D_n (-); b) SEM image diluted latex, bar 1 μm; c) Zeta potential (▲) and D_H (●) as a function of the *pH*; d) AFM film roughness line profile over 6 μm, R_a= 8 nm; e) DSC; f) Storage and loss moduli and loss factor in temperature sweep for cast coatings, dried at 20 °C; g) Molar mass distribution determined with SEC.

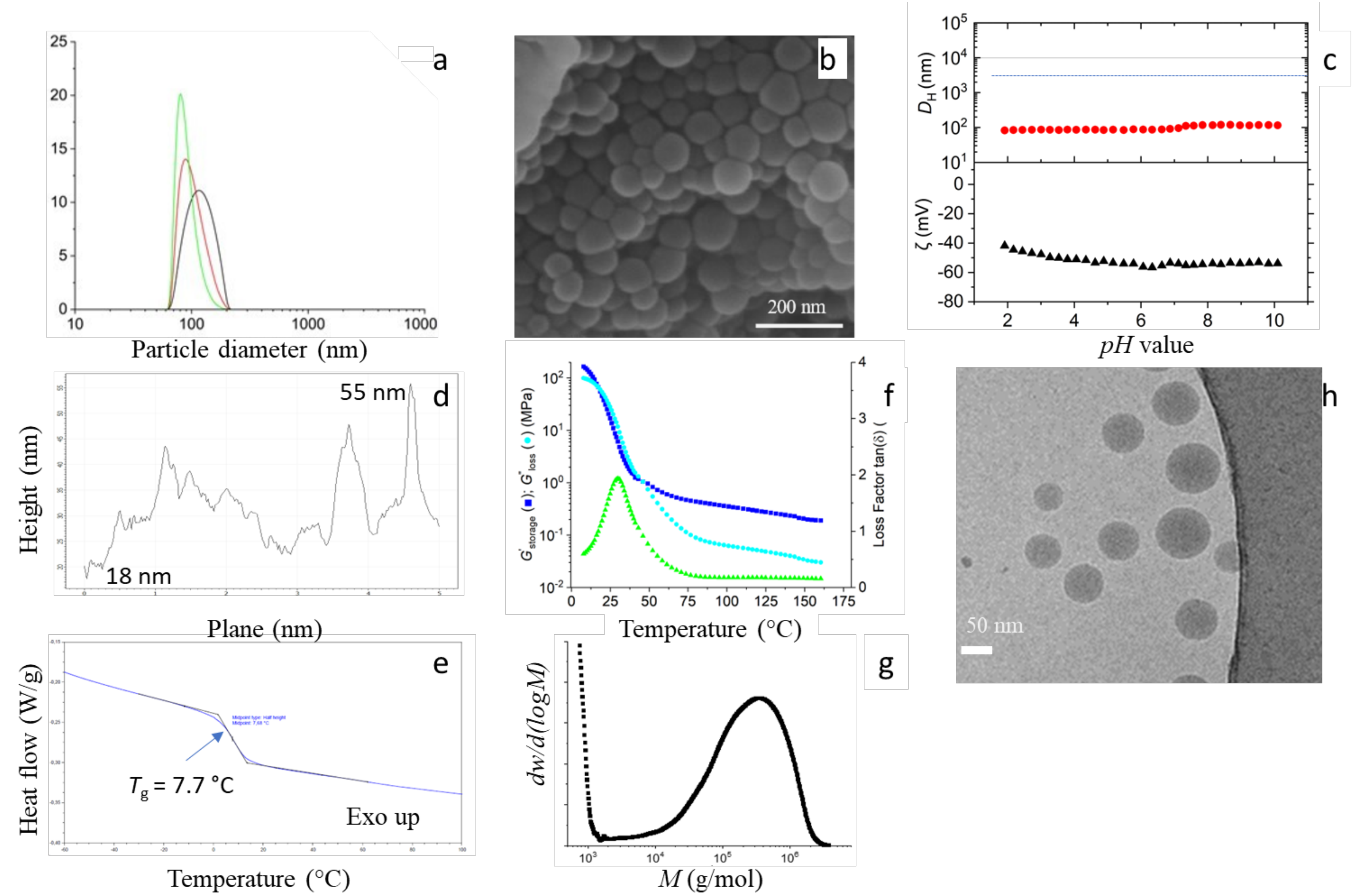

Figure S11 Overview results *L*SDS as surfactant: latex type I, Newtonian; a) PSD CUMULANT model D_I (-); D_v (-) and D_n (-); b) SEM image diluted latex, bar 1 μm; c) Zeta potential (▲) and D_H (●) as a function of the *pH*; d) AFM film roughness line profile over 5 μm, R_a= 9 nm; e) DSC; f) Storage and loss moduli and loss factor in temperature sweep for cast coatings, dried at 20 °C; g) Molar mass distribution determined with SEC h) Cryo-TEM image, bar 50 nm.

4. Comparison of DLS data

Table S2 presents an overview of all averaged particle diameters, including number-averaged (D_n), volume-averaged (D_v), and intensity-averaged (D_I), as well as hydrodynamic diameters (D_H). If multiple peaks are present, the peaks are listed in order of decreasing area. For all latexes the particle size distributions (PSD) are calculated using two different models CUMULANT and CONTIN, only in the case of differences in calculation results both data sets are shown. The applied model in this work is marked with ✓. The measurement threshold of the instrument = 10^4 nm (10 µm).

Table S2 Overview DLS data.

Latex	Type	D_H nm	D_n nm		D_v nm			D_i nm			PDI	Calculation Model	Applied model in paper
*s*L$_l$	III	2130	170	1309	1736	184		1583	200		0.33	CUMULANT- general	✓
		2130	17		20	1862	224	1683	234	25	0.33	CONTIN	
*s*L$_s$	I	144	74		101			157			0.22	CUMULANT- general	✓
*s*B$_l$	II	393	350		397			364			0.07	CUMULANT- general	✓
*s*B$_s$	I	472	268		16039	383		351	15343		0.25	CUMULANT- general	
		467	291		8744	347		342	8862		0.23	CUMULANT- general + sample dialyzed against water	
		472	299		340			337			0.19	CONTIN	✓
*s*M$_l$	I#	192	126		160			196			0.19	CUMULANT- general iso	✓
*s*M$_s$	I#	325	184		3789	263		271	3768		0.22	CUMULANT- general	✓
*b*M	I	119	89		100			117			0.27	CUMULANT- general	✓
*h*A$_l$	II#	>10000	1188		1664			1501			0.40	CUMULANT- general	✓
*h*A$_s$	II#	3355	120		1585	9804	144	907	174	1200	0.24	CUMULANT- general	✓
*L*SDS	I	122	89		110			118			0.11	CUMULANT- general	✓
		122	89		107			134			0.09	CONTIN	

#(some) sedimentation in time

5. AFM images and surface roughness

In Figure S12 and S13 AFM height images (full width is 5 µm) are shown of all coatings using Prep A (dried at 20 °C) and Prep D (dried at 40 °C and annealed at 60 °C for 5 days). In Table S3 the surface roughness (R_a) and the ten-point height (R_z) calculations are shown for all four preparations. The calculations were done over a surface area of 20 x 20 µm and 5 x 5 µm. AFM imaging has been performed within 1 week after aging. In the AFM images in the case of *s*B$_s$, *s*M$_l$, *s*M$_s$ and *h*A$_s$ larger particles can be seen surrounded by smaller particles. Smooth surfaces ($R_a \leq 4$ nm) can be seen for *b*M and *s*L$_s$ (Prep A and D) and *L*SDS (Prep D). The coatings dried at 40 °C are smoother than the ones dried at 20 °C (a coating is considered smooth if $R_a \leq 4$[9]). And after annealing the coatings became even smoother. A higher drying/annealing temperature (above T_g) will improve the interdiffusion/flow of the polymers.

Overview of AFM images

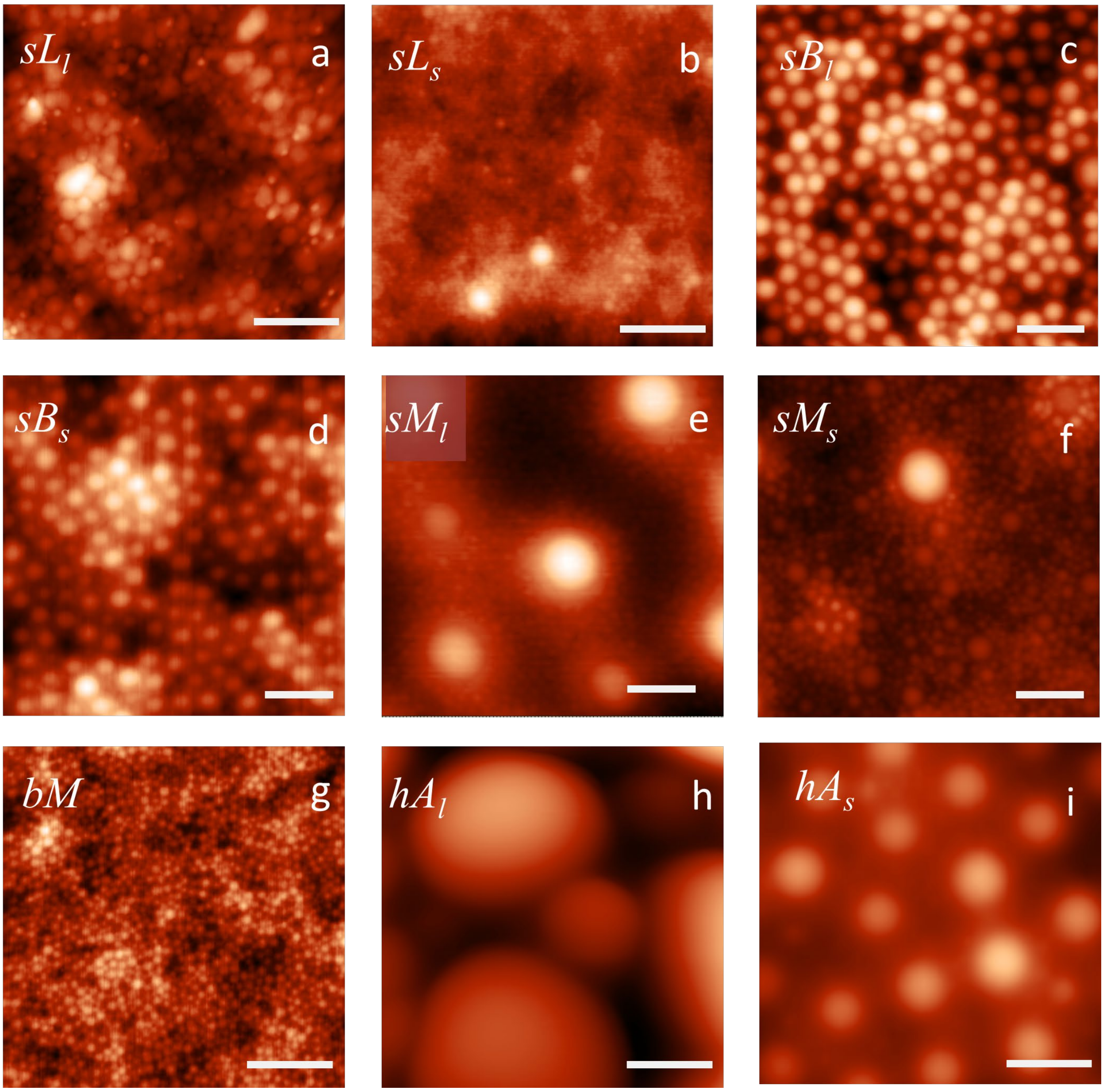

Figure S12 AFM images of coatings made by preparation method A; p(MMA-*co*-BA) latexes synthesized with the following macromonomers: a) *s*-L$_{204\text{-}18}$ b) *s*-L$_{5\text{-}1}$ c) *s*-B$_{297\text{-}34}$, d) *s*-B$_{3\text{-}1}$, e) *s*-M$_{90\text{-}9}$, f) *s*-M$_{30\text{-}10}$, g) *b*-M$_{12\text{-}15}$, h) *h*-MAA$_{350}$, and i) *h*-MAA$_{8}$. Height profile with evaluation length 5 µm, bar 1 µm. For roughness data see Table S3.

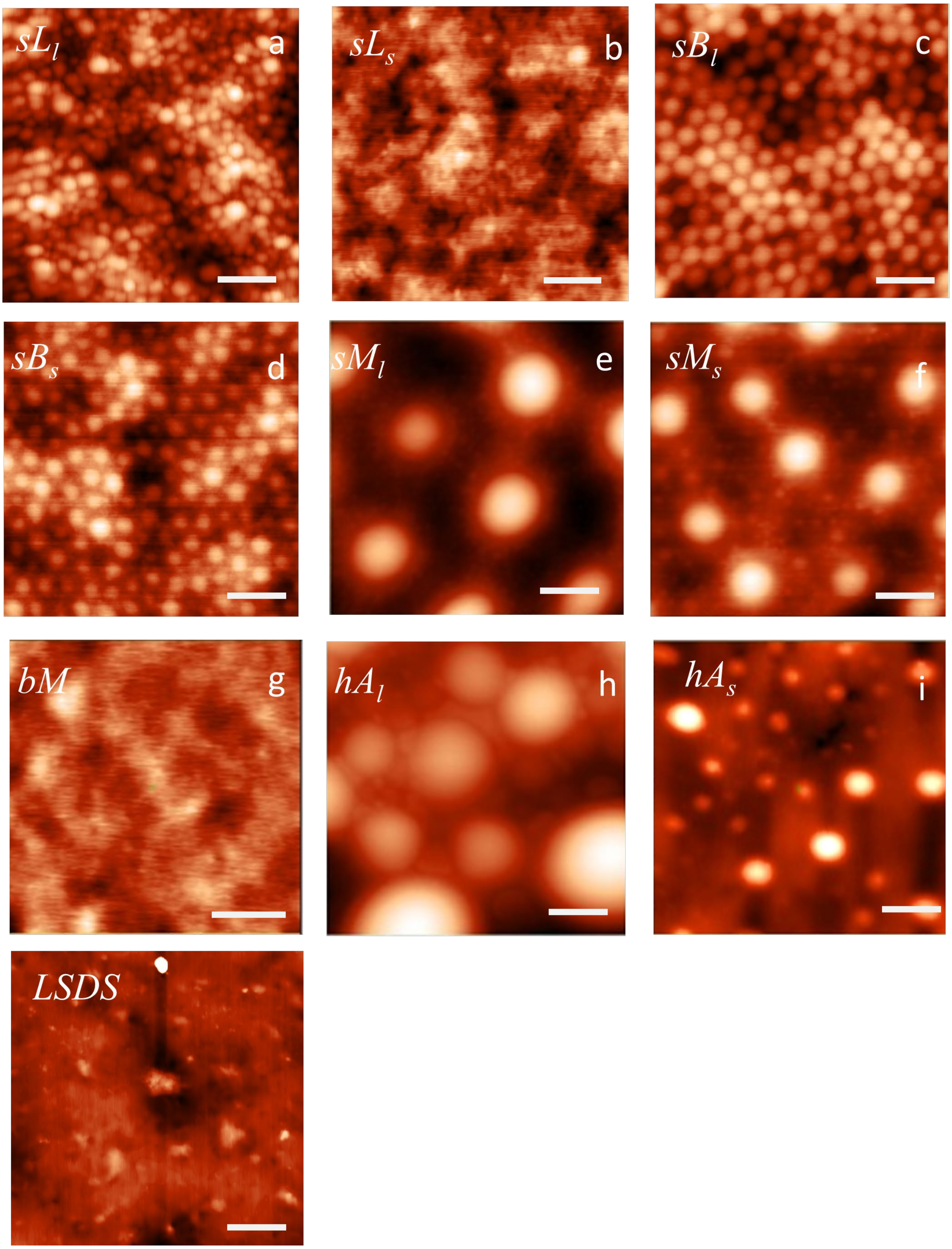

Figure S13 AFM images of coatings made by preparation method D; p(MMA-*co*-BA) latexes synthesized with the following macromonomers a) *s*-L$_{204-18}$ b) *s*-L$_{5-1}$ c) *s*-B$_{297-34}$, d) *s*-B$_{3-1}$, e) *s*-M$_{90-9}$, f) *s*-M$_{30-10}$, g) *b*-M$_{12-15}$, h) *h*-MAA$_{350}$, and i) *h*-MAA$_{8}$, and j) SDS. Height profile with evaluation length 5 µm, bar 1 µm. For roughness data see Table S3.

Comparison of surface roughness

Table S3 Surface roughness characteristics (roughness and ten-point height) of films dried under different conditions[a] measured with AFM at evaluation length of 20 µm and 5 µm, respectively.

Latex	Roughness R_a (nm)							
	no annealing		no annealing		annealed at 60 °C		annealed at 60 °C	
	20 °C (Prep A)		40 °C (Prep B)		20 °C (Prep C)		40 °C (Prep D)	
	20 µm	5 µm	20 µm	5 µm	20 µm	5 µm	20 µm	5 µm
$s\mathrm{L_l}$	190	11	20	10	37	7	40	5
$s\mathrm{L_s}$	4	4	3	3	3	2	3	2
$s\mathrm{B_l}$	12	10	12	9	13	6	9	8
$s\mathrm{B_s}$	16	6	6	4	11	6	7	3
$s\mathrm{M_l}$	27	17	27	14	24	8	22	7
$s\mathrm{M_s}$	16	5	12	6	12	6	11	6
bM	3	2	3	3	8	2	2	1
$h\mathrm{A_l}$	242	144	202	66	128	49	138	63
$h\mathrm{A_s}$	19	8	26	21	30	26	9	6
*L*SDS	14	12	-[b]	-[b]	-[b]	-[b]	9	9
	Ten-point height R_z (nm)							
	no annealing		no annealing		annealed at 60 °C		annealed at 60 °C	
	20 °C (Prep A)		40 °C (Prep B)		20 °C (Prep C)		40 °C (Prep D)	
	20 µm	5 µm	20 µm	5 µm	20 µm	5 µm	20 µm	5 µm
$s\mathrm{L_l}$	601	54	138	39	177	28	209	27
$s\mathrm{L_s}$	47	23	21	13	33	10	18	10
$s\mathrm{B_l}$	120	47	69	34	50	28	41	30
$s\mathrm{B_s}$	70	22	31	18	48	24	34	13
$s\mathrm{M_l}$	126	59	126	69	109	49	85	28
$s\mathrm{M_s}$	85	27	59	44	50	39	46	27
bM	13	11	24	17	63	12	9	7
$h\mathrm{A_l}$	772	461	377	322	481	199	35	242
$h\mathrm{A_s}$	153	58	108	71	90	60	367	35
*L*SDS	125	41	-[b]	-[b]	-[b]	-[b]	49	85

[a]Prep A: film dried at 20 °C, Prep B: film dried at 40 °C, Prep C: film dried at 20 °C and annealed at 60 °C, Prep D: film dried at 40 °C and annealed at 60 °C; [b] For *L*SDS only films using method Prep A and Prep D were measured.

6. Overview of SEM images

In Figure S14 an overview of typical SEM images of diluted latexes (dilution ca 1000x) at comparable magnification is shown. Clearly the difference in particle sizes and particle size distributions can be seen.

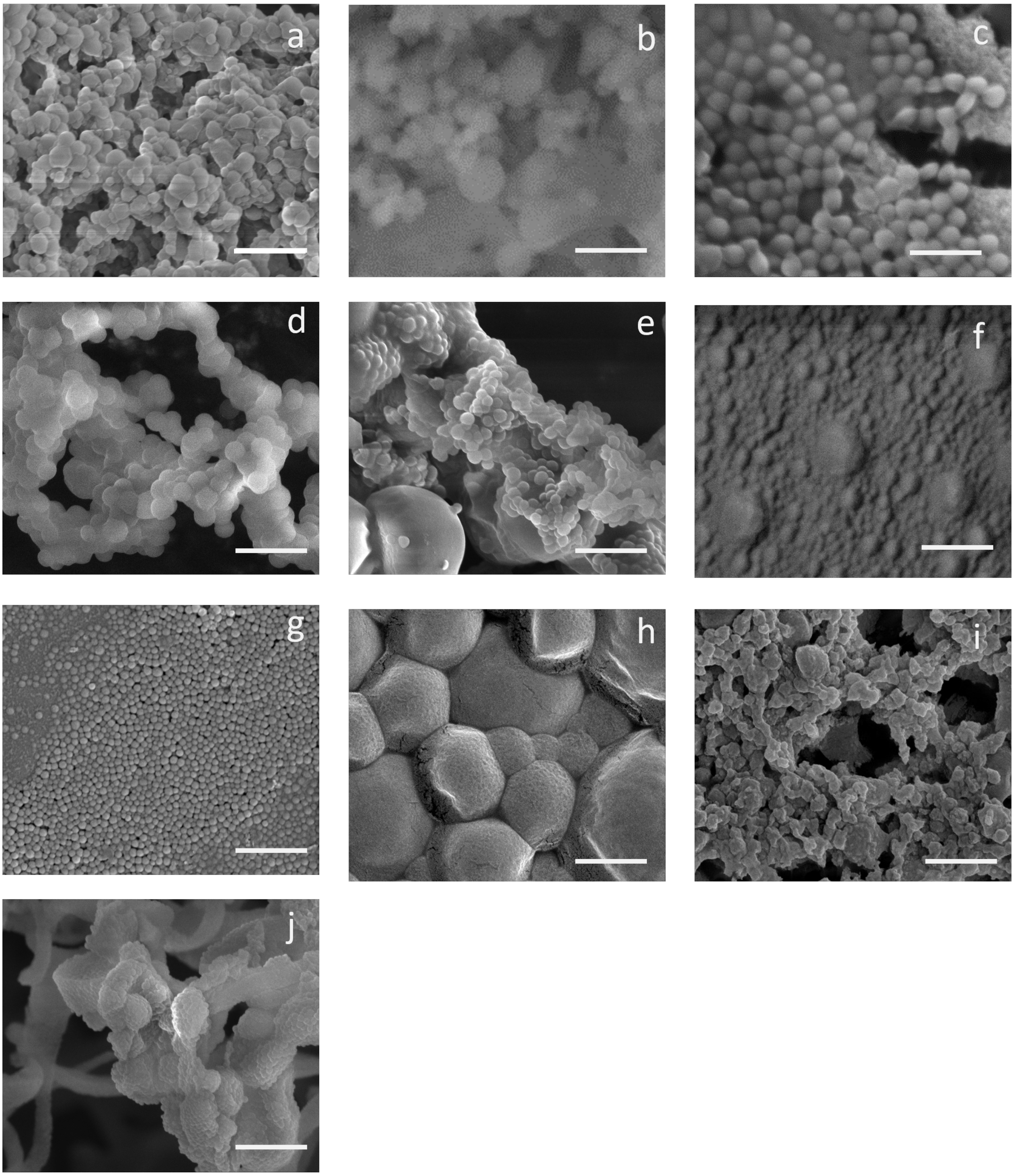

Figure S14 Sem images of all p(MMA-*co*-BA) latexes synthesized with the following macromonomers: a) *s*-L_{204-18}, b) *s*-L_{5-1} (ESEM) c) *s*-B_{297-34}, d) *s*-B_{3-1}, e) *s*-M_{90-9}, f) *s*-M_{30-10}, g) *b*-M_{12-15}, h) *h*-MAA_{350}, and i) *h*-MAA_{8}, j) *L*SDS, bar is 1 µm, the horizontal field width of a full image is comparable with the AFM images with an evaluation length of 5 µm. Bar = 1 µm.

7. Comparison of SEM and Cryo-TEM images

In the following figures comparisons of some SEM and cryo-TEM images are made in case of interesting observations. In Figure S15 we compare cryo-TEM and SEM images of latex sB_l and sB_s, in the cryo-TEM image of sB_l the large hairy structures of the surfactant protruding in the water phase can be seen, the smaller surfactants in the case of sB_s are not visible. In SEM no structures on the surface were visible.

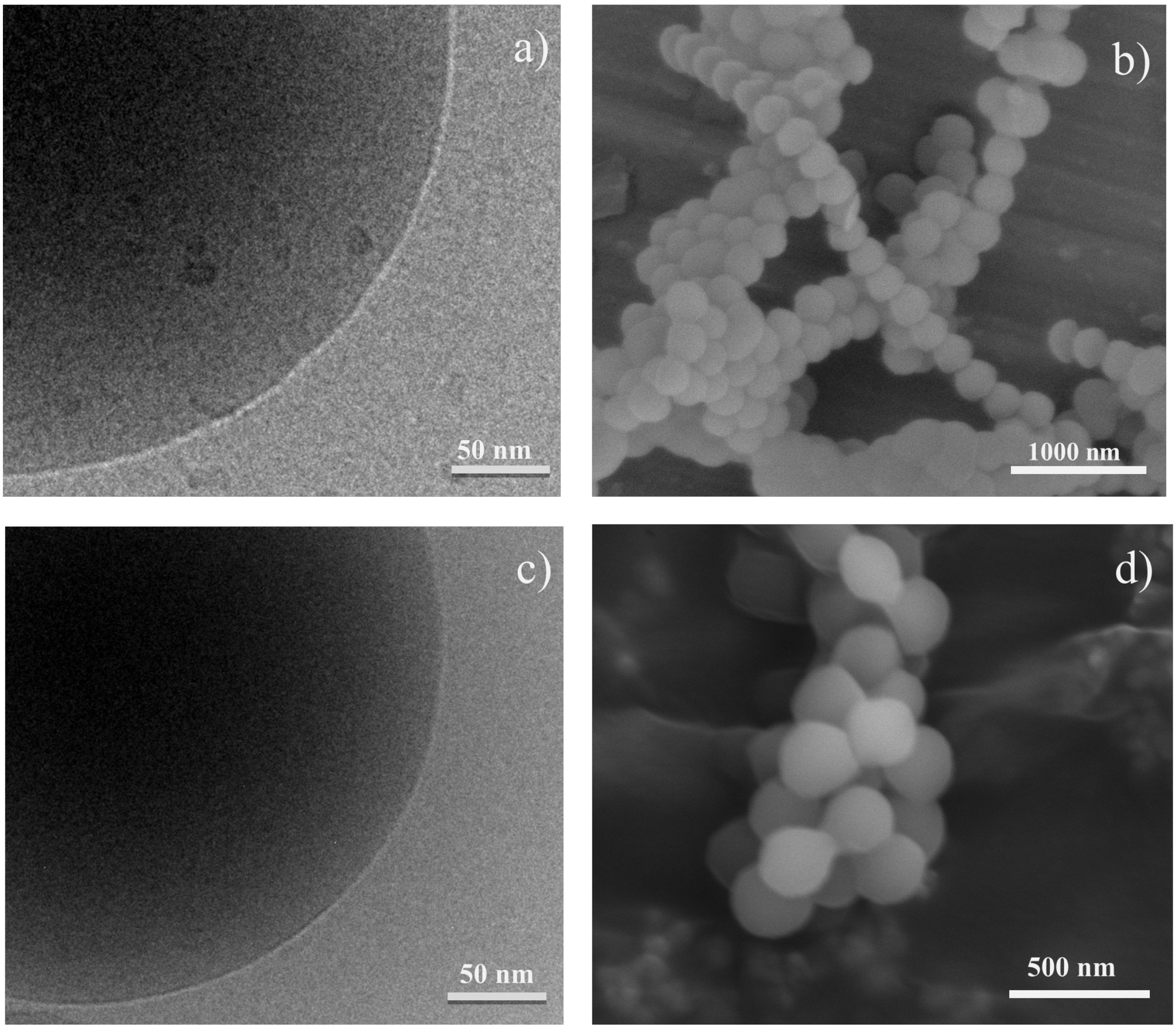

Figure S15. a) Cryo-TEM image and b) SEM image, respectively, of diluted latex sB_l; c) Cryo-TEM image and d) SEM image, respectively, of diluted latex sB_s.

In Figure S16 Cryo-TEM images of vesicle-like (Figure S16a) and more core-shell like (Figure S16b) particles of latex *b*M are shown. The vesicles were only found occasionally and their size is comparable with the other particles. The composition of the vesicles is unknown, but probably formed by the macromonomeric blockcopolymer (*i.e.*, MAA_{12}-MMA_{15}), and subsequently polymerized.

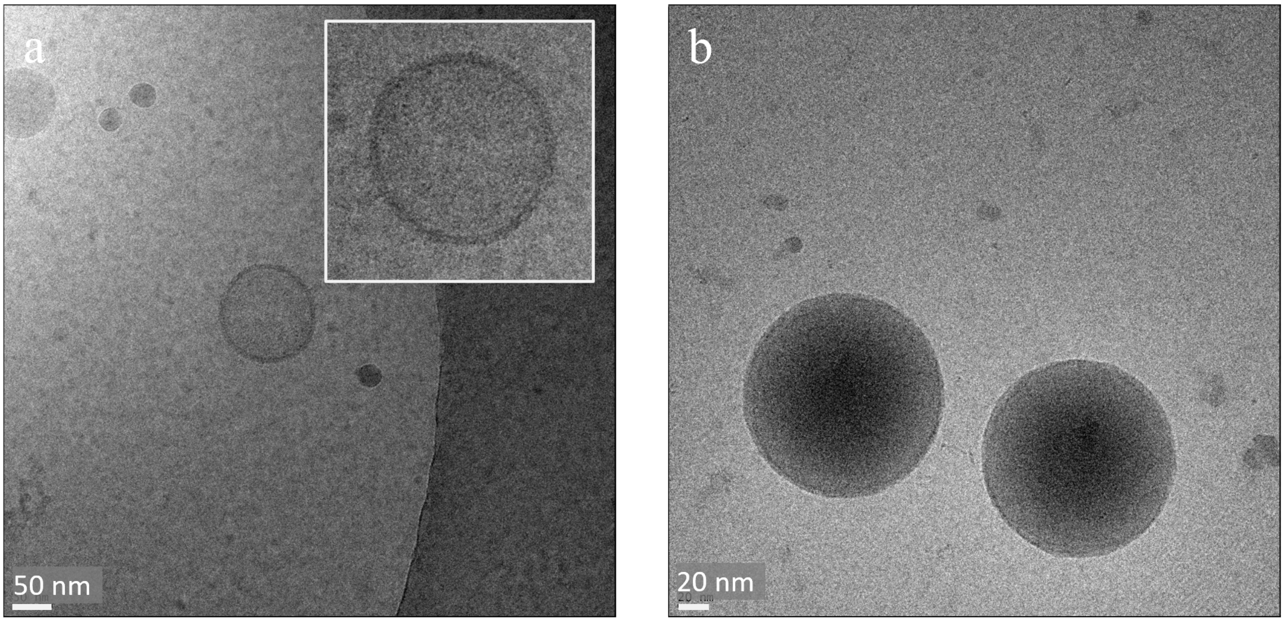

Figure S16 Cryo-TEM images latex b*M* a) vesicles (~170 nm) present b) more core-shell-like particles (~150 nm)

8. Rheology data

In Figure S17 the viscosity (a) and shear stress (b) as a function of the shear rate are shown for all latexes. In Figure S18 the dynamic moduli *G*' and *G*" are recorded for a dynamic time sweep of 1000 seconds, to check the recoverability of the structure, the sample was stirred (using a shear rate of 100 s^{-1} for a duration of 100 s), after which another dynamic time sweep was recorded. A condensed version of Figure S17a and S18 are also plotted in the main paper as Figure 5a and 5b.

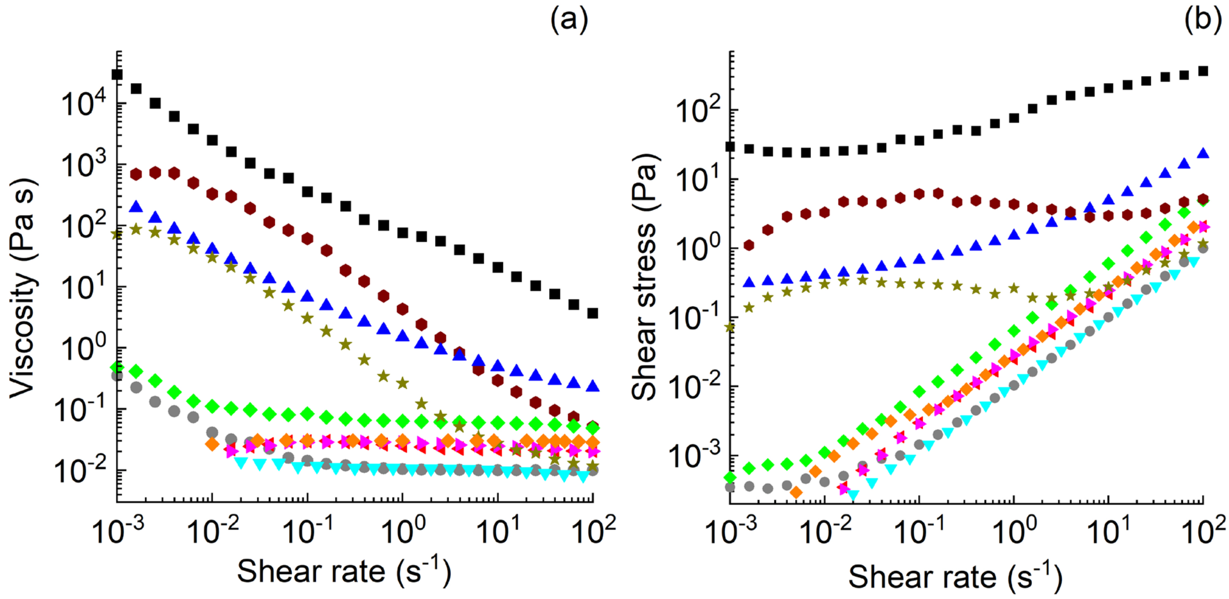

Figure S17 Rheology of p(MMA-*co*-BA) latexes: (a) viscosity and (b) shear stress as a function of shear rate; (■) *s*L_l; (●) *s*L_s; (▲) *s*B_l; (▼) *s*B_s; (◀) *s*M_l; (▶) *s*M_s; (◆) *b*M; (⬢) *h*A_l; (★) *h*A_s; (◆) *L*SDS.

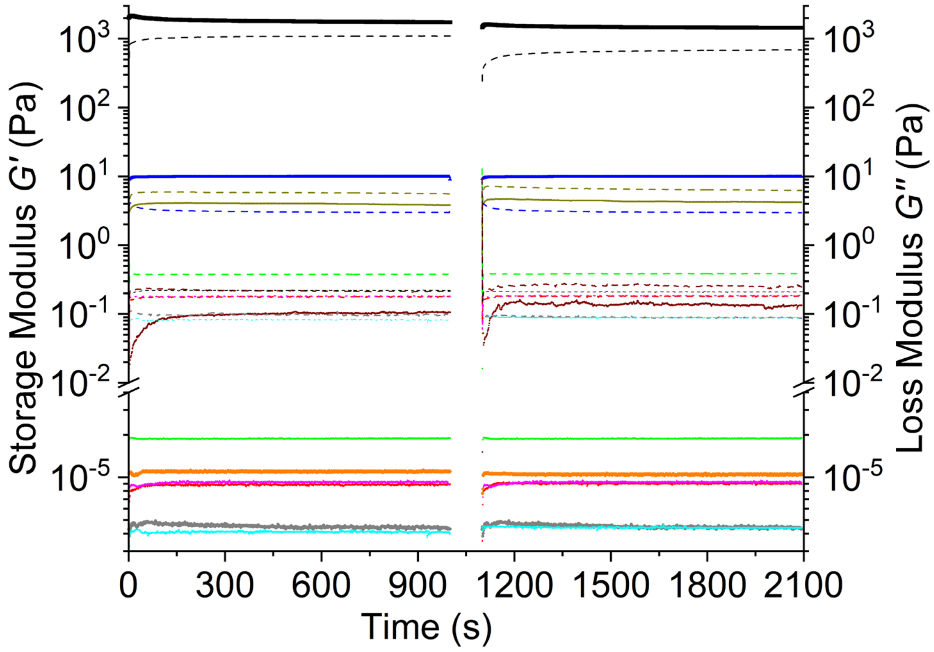

Figure S18 Rheology of p(MMA-*co*-BA) latexes: storage modulus *G*' (solid line) and loss modulus *G*" (dotted line) as function of time: (■) *s*L_l; (●) *s*L_s; (▲) *s*B_l; (▼) *s*B_s; (◀) *s*M_l; (▶) *s*M_s; (◆) *b*M; (⬢) *h*A_l; (★) *h*A_s; (◆) *L*SDS.

9. Overview of static and dynamic contact angle measurements

In Table S4 the water contact angle measurements (static and dynamic) for all coatings are shown. After annealing all coatings became more hydrophobic.

Table S4. Static and dynamic contact angles of coating dried at different conditions.[a]

Polymer film from latex	Contact angle				advancing contact angle (ACA) and receding contact angle (RCA)							
	no annealing		annealed at 60 °C		no annealing				annealed at 60 °C			
	20 °C (Prep A)	40 °C (Prep B)	20 °C (Prep C)	40 °C (Prep D)	20 °C (Prep A)		40 °C (Prep B)		20 °C (Prep C)		40 °C (Prep D)	
					ACA	RCA	ACA	RCA	ACA	RCA	ACA	RCA
*s*L$_l$	71	71	96	88	86	11	75	13	92	13	87	16
*s*L$_s$	61	55	68	70	59	8	59	21	72	28	62	26
*s*B$_l$	48	51	85	60	55	13	58	na[b]	82	na[b]	56	na[b]
*s*B$_s$	36	11	26	21	28	na[b]	26	na[b]	<10	-	<10	-
*s*M$_l$	67	77	76	<10	77	11	86	20	82	14	<10	-
*s*M$_s$	52	58	55	55	50	17	<10	-	51	8	47	20
*b*M	69	71	78	81	76	8	79	20	86	47	81	23
*h*A$_l$	65	56	76	67	57	18	45	15	52	18	51	16
*h*A$_s$	21	57	70	81	52	11	50	13	74	25	83	10
*L*SDS	47	51	61	60	50	7	54	26	57	16	70	27

[a]Prep A: film dried at 20 °C, Prep B: film dried at 40 °C, Prep C: film dried at 20 °C and annealed at 60 °C, Prep D: film dried at 40 °C and annealed at 60 °C, drying is overnight and annealing for 5 days; [b]No lower plateau value reached for RCA.

10. Comparison of DVS sorption plots

In Figure S19 the water sorption as a function of partial water pressure (relative humidity = *RH*) for emulsion copolymers is shown. The relative increase of the sample mass (black lines) is measured with increasing *RH* (red lines). The *RH* was increased from 0% to 95% and back from 95% to 0% in steps of 5-10 % in time. The large increase of the water uptake of *s*B$_{s}$ above *RH* > 60% is clearly visible, this is ascribed to hydroplasticization. Figure S20 shows a typical example of moisture vapor measurement (*i.e.* *s*L$_{s}$) using the Payne cell.

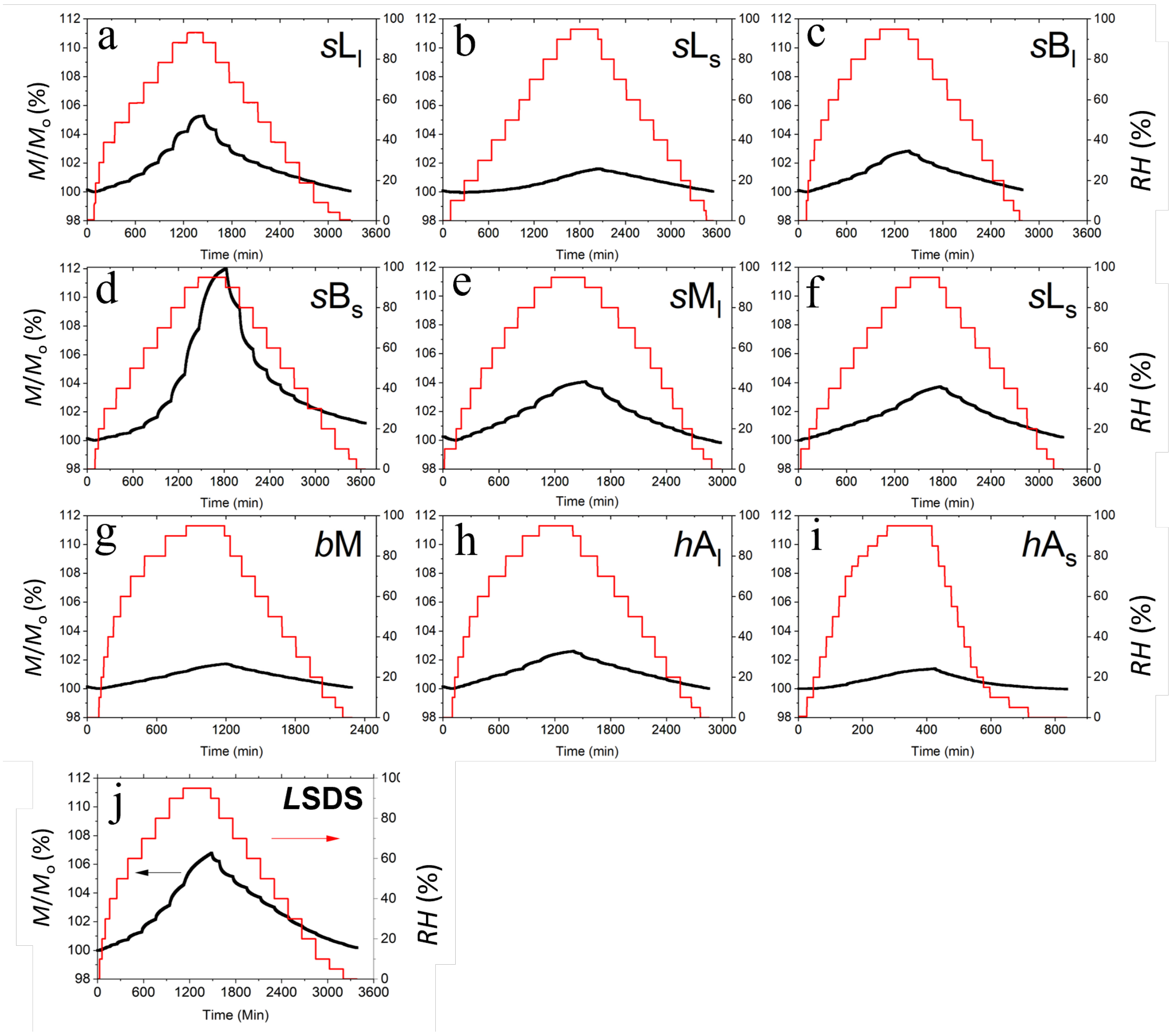

Figure S19 Water sorption (black lines) of p(MMA-*co*-BA) film as a function of partial water pressure (red lines) by DVS. Copolymers synthesized with the following macromonomers via emulsion polymerization: a) *s*-L$_{204-18}$, b) *s*-L$_{5-1}$, c) *s*-B$_{297-34}$, d) *s*-B$_{3-1}$, e) *s*-M$_{90-9}$, f) *s*-M$_{30-10}$, g) *b*-M$_{12-15}$, h) *h*-MAA$_{350}$, i) *h*-MAA$_{8}$, and j) with SDS as surfactant.

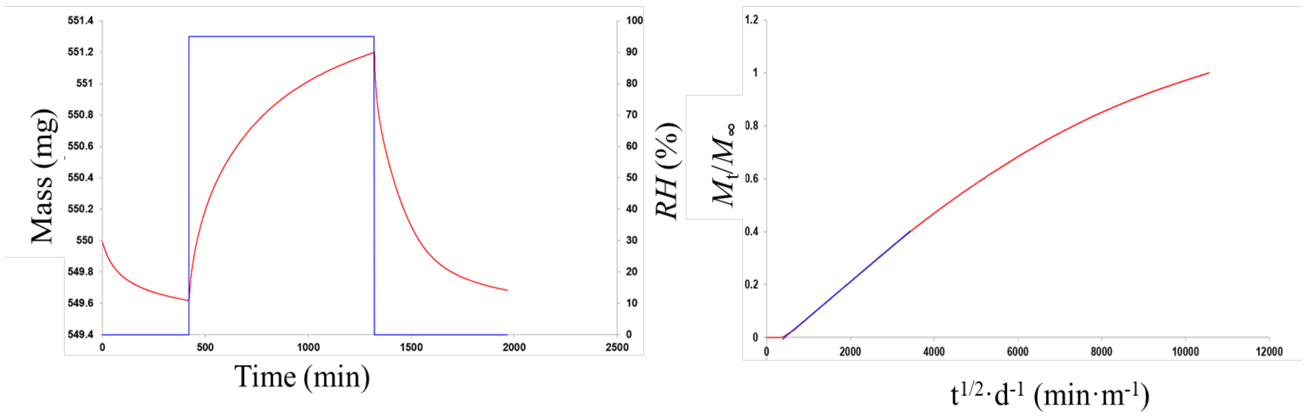

Figure S20 DVS analysis of $s\mathrm{L}_s$ coating using a Payne cell; a) Mass (red curve) and relative humidity (blue curve) versus time; b) M_t/M_∞ (red curve) versus $t^{1/2}$ d^{-1}, the blue line indicates the slope used to calculate D_{diff}.

11. Additional results of emulsion polymerization

In Figure S21 a plot is shown for the first hour of the emulsion polymerization.

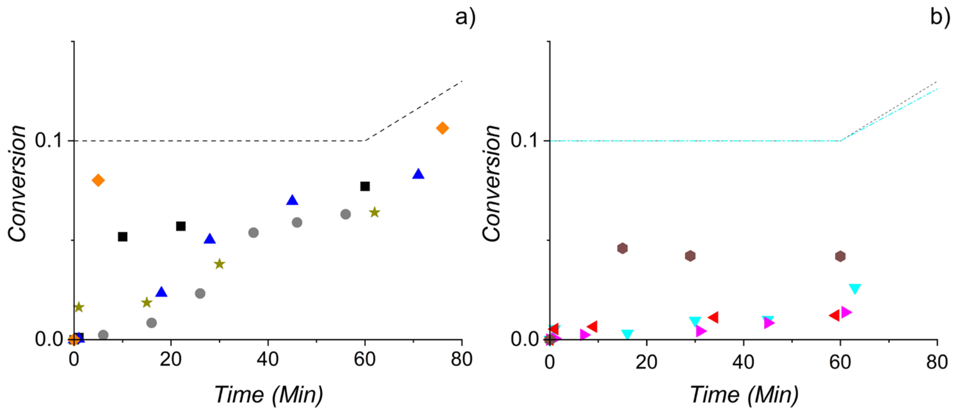

Figure S21 Overall monomer conversion versus time curves for the semi-batch emulsion copolymerization of BA and MMA (1:1 w/w) with different stabilizers: a) (■) s-$\mathrm{L}_{204\text{-}18}$; (●) s-$\mathrm{L}_{5\text{-}1}$; (▲) s-$\mathrm{B}_{297\text{-}34}$; (◆) b-$\mathrm{M}_{12\text{-}15}$; (★) h-MAA_8 and (◆) SDS with feed rate of 5 mL h^{-1}; b) (◀) s-$\mathrm{M}_{90\text{-}9}$; (▶) s-$\mathrm{M}_{30\text{-}10}$, (⬢) h-MAA_{350} and with feed rate of 5 mL h-1 and (▼) s-B3-1 with feed rate of 4 mL h^{-1}. Initial charge of monomer 9 wt%, seed time 1 h; remaining 91 wt% fed (dashed line).

All latexes except for *L*SDS have been centrifuged and the supernatant was freeze dried. In five cases ($s\mathrm{B}_s$, $s\mathrm{M}_l$, $s\mathrm{M}_s$, $h\mathrm{A}_l$ and $h\mathrm{A}_s$) some polymer was obtained and analyzed using SEC. The results are shown in Figure S22. The other latexes did not contain analyzable amounts of polymer. These results are consistent with the decrease in surface tension of a water droplet from the coating surface (see Table 5, main text).

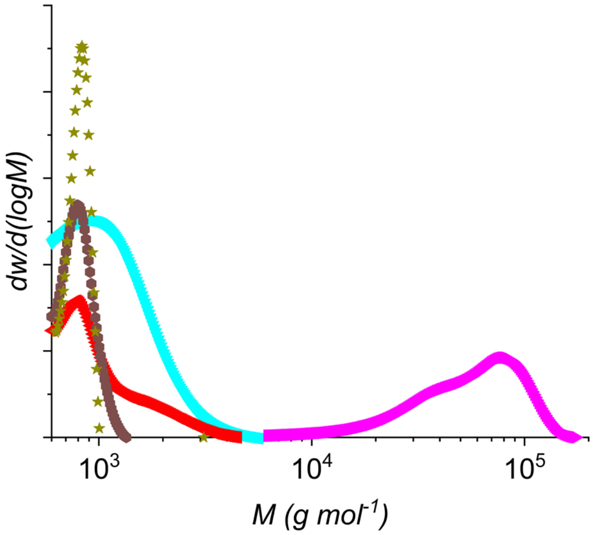

Figure S22 Molar mass distribution of (freeze-dried) supernatant polymer of centrifuged latex determined using SEC, (▼) *s*B$_s$; (◀) *s*M$_l$; (▶) *s*M$_s$; (⬢) *h*A$_l$ and (★) *h*A$_s$, areas not normalized for concentration of polymer present in supernatant.

References

(1) Andrews, R. J.; Grulke, E. A. *Polymer Handbook*, 4th ed.; Brandrup J, Immergut E. H.; Grulke E. A., (Eds.); Wiley: New York, 2003.

(2) ISO 21920-2:2020, Geometrical Product Specifications (GPS) — Surface Texture: Profile — Part 2: Terms, Definitions, and Surface Texture Parameters.

(3) *Standard Test Methods for Measuring Adhesion by Tape ASTM D3359*.

(4) Huhtamäki, T.; Tian, X.; Korhonen, J. T.; Ras, R. H. A. Surface-Wetting Characterization Using Contact-Angle Measurements. *Nat Protoc* **2018**, *13* (7), 1521–1538.

(5) Berry, J. D.; Neeson, M. J.; Dagastine, R. R.; Chan, D. Y. C.; Tabor, R. F. Measurement of Surface and Interfacial Tension Using Pendant Drop Tensiometry. *J Colloid Interface Sci* **2015**, *454*, 226–237.

(6) *Standard Test Methods for Hardness of Organic Coatings by Pendulum Damping Tests ASTM D4366*; 2016.

(7) *Standard Test Method for Specular Gloss ASTM D523-14*; 2018.

(8) Crank J.J.; Park G.S. (Eds.). *Diffusion in Polymers*, 2nd ed.; Academic Press: London, 1975.

(9) Butt, H.-J.; Gerharz, B. Imaging Homogeneous and Composite Latex Particles with an Atomic Force Microscope. *Langmuir* **1995**, *11*, 4735–4741.
